# A platinum-based hybrid drug design approach to circumvent acquired resistance to molecular targeted tyrosine kinase inhibitors

**DOI:** 10.1038/srep25363

**Published:** 2016-05-06

**Authors:** Yuming Wei, Daniel C. Poon, Rong Fei, Amy S. M. Lam, Steve C. F. Au-Yeung, Kenneth K. W. To

**Affiliations:** 1 Department of Chemistry, Faculty of Science, The Chinese University of Hong Kong, Hong Kong SAR, China; 2 School of Pharmacy, Faculty of Medicine, The Chinese University of Hong Kong, Hong Kong SAR, China

## Abstract

Three molecular targeted tyrosine kinase inhibitors (TKI) were conjugated to classical platinum-based drugs with an aim to circumvent TKI resistance, predominately mediated by the emergence of secondary mutations on oncogenic kinases. The hybrids were found to maintain specificity towards the same oncogenic kinases as the original TKI. Importantly, they are remarkably less affected by TKI resistance, presumably due to their unique structure and the observed dual mechanism of anticancer activity (kinase inhibition and DNA damage). The study is also the first to report the application of a hybrid drug approach to switch TKIs from being efflux transporter substrates into non-substrates. TKIs cannot penetrate into the brain for treating metastases because of efflux transporters at the blood brain barrier. The hybrids were found to escape drug efflux and they accumulate more than the original TKI in the brain in BALB/c mice. Further development of the hybrid compounds is warranted.

Tyrosine kinase inhibitors (TKIs) are an important new class of molecular targeted chemotherapeutic drugs that specifically inhibit oncogenic tyrosine kinases to kill cancer cells by regulating cancer proliferation, invasion, metastasis and angiogenesis. Unfortunately, while TKIs are highly effective against CML and other solid tumors associated with deregulation of kinase pathways, their usefulness is severely compromised by drug resistance mediated by various mechanisms^1^,^2^. Among them, the emergence of point mutations of the target kinase at the drug-kinase-interaction domain is the most commonly observed^3^. To overcome TKI resistance mediated by these mutations, new generations of TKIs have been developed by structural modification of existing functional groups on the original drug backbone to restore binding to the mutated TK either reversibly or irreversibly^4^,^5^,^6^. However, the 2^nd^ generation irreversible TKI (e.g., afatinib and dacomitinib) are not sufficiently effective against the resistant cells at a clinically achievable concentration *in vivo* due to toxicity and/or limited bioavailability^7^,^8^. Rational combinations of TKIs with inhibitors of other downstream/parallel signaling pathways have also been studied with an aim to circumvent resistance^9^. However, none of these approaches has been successfully adopted for clinical use. Most recently, the 3^rd^ generation mutant selective TKIs have been developed^10^,^11^. They are promising drug candidates for further development because they are effective against tumors bearing both sensitizing and resistance mutation. Importantly, they were also found to spare the wild type oncogenic receptor, thus exhibiting a better adverse effect profile than the 2^nd^ generation TKIs.

In this study, we exploited our previously established hybrid drug design platform (Platinum (Pt) drug core+ Bioactive Ligand^12^) to generate nine Pt-TKI hybrid compounds with an aim to circumvent TKI resistance, by incorporating one of three TKIs (imatinib, erlotinib and vandetanib, respectively) into the core structure of Pt-based anticancer drugs (cisplatin, oxaliplatin or transplatin). Pt has high reactivity to nucleophilic nitrogen; akin to the formation of DNA-Pt adduct, Pt reacts readily with the nitrogen atom on the quinazoline ring (erlotinib and vandetanib) and pyrimidine ring (imatinib) of the TKIs to form the hybrids. Cisplatin and oxaliplatin are clinically approved anticancer drugs. Transplatin is not used in the clinic but its different ligand binding configuration (“trans” in transplatin versus “cis” in cisplatin/oxaliplatin) allows us to study the effect of stereochemistry on various properties of the new compounds.

To this end, TKIs (including erlotinib, gefitinib, imatinib and sunitinib) have been covalently linked to lysozyme via a Pt-based moiety (termed universal linkage system (ULS)) for organ/cell type specific delivery of the molecular targeted drugs to angiogenic endothelial cells, hepatic stellate cells and kidney tubular cells^13^. However, these drug conjugates were not intended for use in cancer chemotherapy. The salient properties of these conjugates are their stability *in vivo* and the feasibility for sustained drug release within the target cells.

In this paper, we report on the circumvention of TKI resistance, mediated by the secondary mutation of the oncogenic kinases, by the Pt-TKI hybrids *in vitro.* The hybrids were found to maintain specificity towards the same oncogenic kinase as the original TKI. However, they were shown to bind to a slightly different site(s) in the ATP binding pocket of the kinases, thus allowing them to be less affected by the secondary mutation. Moreover, the observed dual mechanisms of action exhibited by the hybrid compounds, which include kinase inhibition and monofunctional DNA platination, may also explain the partial relief of drug resistance. Furthermore, most TKIs are substrates of multidrug resistance (MDR) transporters, which effectively pump the drugs out of cancer cells. Since Pt drugs are not substrates of MDR transporters, we also intend to exploit the hybrid drug design approach to turn the TKI into non-substrate so as to avoid the transporters-mediated drug resistance and other related pharmacokinetic problems.

## Results

### Chemistry

**Figure 1:**
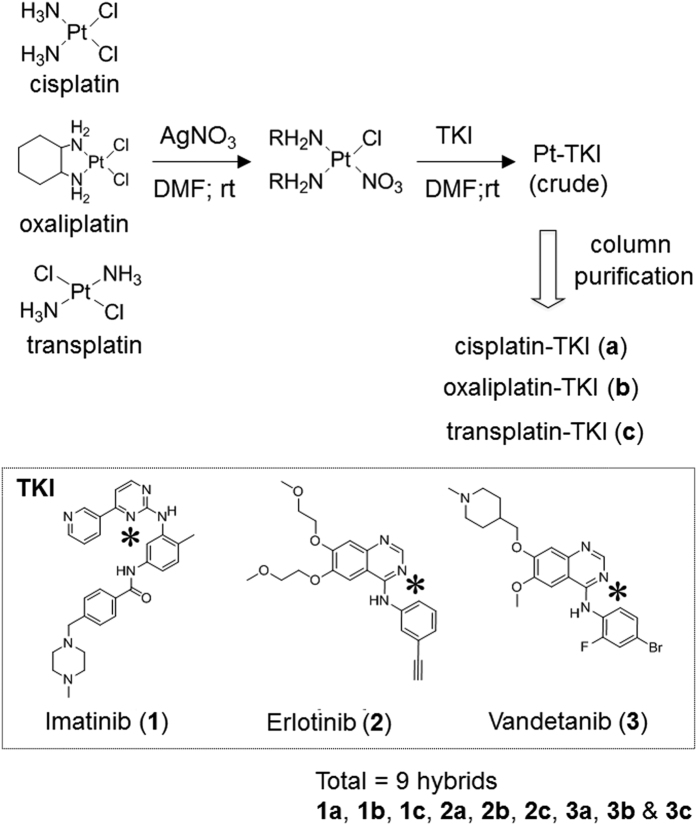
Synthetic scheme of the three series of Pt-TKI hybrids. Imatinib, erlotinib or vandetanib were conjugated through a nitrogen atom (labeled *) on their pyrimidine (for imatinib) or quinazoline (for erlotinib and vandetanib) ring to the Pt core atom of cisplatin, oxaliplatin or transplatin. **1a** Cisplatin-imatinib; **1b** Oxaliplatin-imatinib; **1c** Transplatin-imatinib; **2a** Cisplatin-erlotinib; **2b** Oxaliplatin-erlotinib; **2c** Transplatin-erlotinib; **3a** Cisplatin-vandetanib; **3b** Oxaliplatin-vandetanib; **3c** Transplatin-vandetanib. DMF = dimethylformamide; rt = room temperature.

The synthesis of the hybrid compounds (**1a–1c**, **2a–2c**, **3a–3c**) is described in Fig. 1. Three TKIs (imatinib, erlotinib, vandetanib) were individually conjugated to Pt-based anticancer agents (cisplatin, oxaliplatin and transplatin), respectively, according to a published method with minor modifications^14^. Briefly, the Pt compounds were allowed to react with AgNO_3_ in equal molar ratio in dimethylformamide for 8 h at room temperature to facilitate the replacement of one Cl with a NO_3_ group. The Pt nitrate product was obtained after filtration. Equal molar TKI (free base form) was then added to the Pt nitrate solution to produce the Pt-TKI hybrid crude products. Solvents were evaporated under reduced pressure. The crude hybrid compounds were further purified by size-exclusion (Sephadex LH-20, GE Healthcare) or silica gel (C-18) column chromatography using dimethylformamide or 0.1% trifluoroacetic acid in water/acetonitrile gradient as the mobile phase, respectively. The purified hybrid compounds were fully characterized by ^1^H NMR, ^14^N NMR, ^13^C-^1^H HSQC, ^1^H-^1^H delayed COSY and high resolution mass spectrometry. The product was confirmed to be the desired Pt-TKI hybrid with the Pt moiety and TKI in 1:1 stoichiometry. Their purity range from 91–99% as evaluated by HPLC. Representative characterization data can be found in the supporting information (Supplementary Fig. S1a–k).

The chemical stability of the hybrid compounds were evaluated in saline (0.9% sodium chloride), phosphate buffered solution (pH 4 and 7.4), or culture medium (Dulbecco’s Modified Eagle Medium supplemented with 5 mM glutathione) mimicking the intracellular compartment. While all hybrids are stable in saline and phosphate buffered solutions at 37 °C over 24 h, the transplatin-based hybrids (Transplatin-Imatinib (**1c**) and Transplatin-Erlotinib (**2c**)) were found to undergo gradual degradation to release the original TKI in culture medium (Supplementary Fig. S2). On the other hand, the other hybrids (**1a**, **1b**, **2a**, **2b**, **3a**–**3c**) were all found to be stable in all conditions tested. The breakdown of the transplatin-TKI hybrids into their constituent Pt moiety and TKI is likely due to the strong trans-effect from a sulfur-containing biomolecule ligand (e.g. glutathione), which displace the TKI from the Pt coordination core.

### Selectivity Profile

**Figure 2:**
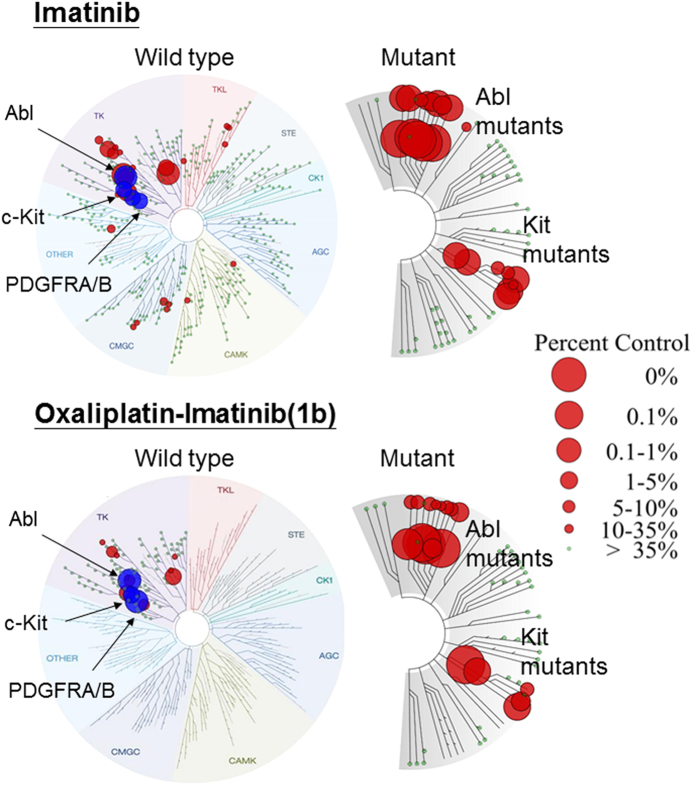
TREE*spot* compound profile data (DiscoveRx) summarizing the kinase inhibition of 132 oncogenic kinases by imatinib and 1b (oxaliplatin-imatinib). Kinase inhibition profiling was performed by the KINOME*scan* service (DiscoveRx). The inhibition of different kinases is expressed as percentage of control and it is labeled in circle. The bigger the circle the greater is the inhibition effect. Both imatinib and **1b** share similar kinase selectivity. They are selective towards Abl, c-kit and PDGFR (labeled blue on the diagram).

The Pt-TKI hybrids were first evaluated for their selectivity profile on different oncogenic kinases. Kinase inhibition profiling was conducted on a panel of 132 kinases upon incubation with 10 μM of the hybrids (DiscoveRx Corporation, Fremont, CA, USA; Fig. 2, and Supplementary Fig. S3 and Supplementary Table S1 in the Supporting Information). By Spearman rank order correlation analysis, representative hybrids from each of the three series (Pt-erlotinib, Pt-imainib, or Pt-vandetanib) were found to share highly similar spectrum of kinase inhibition with their original TKI (rho = 0.89 (*p* < 0.001), rho = 0.61 (*p* < 0.001), rho = 0.83 (*p* < 0.001), **1b**, **2a**, **3b**, respectively). This shows that the hybrid design did not change the kinase inhibition selectivity of the TKI. Importantly, this is reflected by the much greater potency of the Pt-erlotinib and Pt-vandetanib hybrids in HCC827 (harboring the sensitizing EGFR ΔE746-A750 mutation) than in H292 (harboring wild-type EGFR); and of the Pt-imatinib hybrids in K562 (Bcr-Abl^+^) than in RPMI8226 (Bcr-Abl^−^) (Table 1). The hybrid compounds are unlikely to cause side effect by inhibiting normal cells without the oncogenic abnormalities. The toxicity of the hybrids in a normal porcine kidney epithelial cell line LLC-PK1 was also evaluated. This was to examine if the Pt moiety in the hybrid would confer nephrotoxicity to the hybrids. In contrast to the remarkable concentration-dependent nephrotoxicity demonstrated by cisplatin, all hybrid exhibits minimal toxicity to the normal kidney cells (Supplementary Fig. S4).

### Circumvention of TKI resistance mediated by specific oncogenic mutation

**Figure 3:**
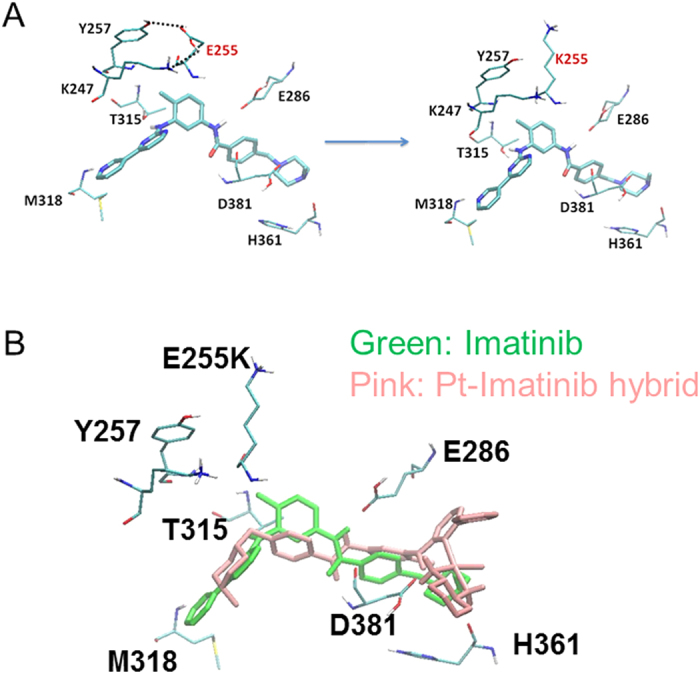
(**A**) Structure information of Bcr Abl E255K illustrating why this mutant affects binding of imatinib. E255 is located in the P loop of the Abl kinase domain. It interacts strongly with Y257 and K247 (left diagram) and facilitates the fitting of imatinib into the binding pocket. Mutation E255K destabilizes the P-loop conformation and it adversely affects the binding of imatinib (right diagram). (Shah *et al.*). (**B**) According to docking simulation, a representative Pt-Imatinib hybrid **1b** binds with a slightly different conformation (compared with imatinib) to the Abl binding pocket. It is less affected by the Bcr Abl E255K mutation.

The three series of Pt-TKI hybrids were evaluated in resistant cell models bearing specific secondary mutation of the oncogenic kinases or induced by prolonged drug exposure. Among the three series of Pt-TKI hybrids, the Pt-imatinib ones (**1a**, **1b**) were found to be the least affected by TKI resistance. It has been reported that E255K is one of the two major mutations in Bcr/Abl contributing to imatinib resistance *in vitro* and in patient specimens^15^. Bcr-Abl E255K mutation destabilizes the P-loop conformation of the key imatinib binding residues (i.e. T315 and M318)^16^. By docking simulation, the three Pt-imatinib hybrids (as illustrated by **1b**) interact with Bcr-Abl E255K mutant (PDB ID code 1IEP, with E255 changed to K) at a slightly different position of the ATP-binding pocket of Abl and are less relying on T315 and M318 (Fig. 3). **1b** is estimated to form H-bond with D381, H361 and R362 (Supplementary Table S1). The slightly altered binding site may minimize the influence of E255K mutation on the Pt-Imatinib hybrids, and allow them to escape TKI resistance. Importantly, this is supported by data from the cytotoxicity assay. While imatinib was more than 15 times less potent in HEK293 cells stably transfected with Bcr/Abl E255K mutant than in the wild type Bcr/Abl cells, the anticancer activity of the Pt-imatinib hybrids were not significantly affected (Table 2). Moreover, this is also consistent with data from the cell-free biochemical assay evaluating the Bcr-Abl kinase inhibition by the Pt-Imatinib hybrids. For imatinib, its IC_50_ in inhibiting Bcr-Abl E255K (>10000 nM) is more than 364 times greater than that in the Bcr-Abl wild type kinase (Table 3). In contrast, the IC_50_ of the Pt-Imatinib hybrids in inhibiting Bcr-Abl E255K is only 5–10 times higher than that in the wild type Bcr-Abl (Table 3).

Similar circumvention of TKI resistance by Pt-Erlotinib and Pt-Vandetanib hybrids in NSCLC cell line (H1975) harboring the most prevalent resistance-causing secondary mutation (EGFR T790M) were also observed, but to a lesser extent (Table 1). This is also consistent with the much less affected kinase inhibitory effect of the EGFR L858R/T790M mutant kinase by the Pt-Erlotinib and Pt-Vandetanib hybrids, relative to EGFR L858R (Table 3). Importantly, similar to the case of Pt-imatinib hybrids, this is consistent with the docking simulation analysis where Pt-Erlotinib and Pt-Vandetanib hybrids interact with the EGFR T790M mutant at slightly different sites in the ATP binding pocket and are thus less affected by the mutation (Supplementary Fig. S5).

### Further evidence to demonstrate the altered binding site(s) of hybrids on the oncogenic kinase

Docking simulation was used not only to model the interaction of the Pt-TKI hybrids to EGFR or Bcr-Abl *in silico*, respectively, but also to predict interacting amino acid residues in the oncogenic kinases that could be mutated to provide a definitive proof of the interaction. To specifically establish the binding of Pt-TKI hybrids to an altered site in the kinase domain of the EGFR and Bcr-Abl, we created EGFR T790M and Bcr-ABl E255K constructs in which a predicted additional interaction site by the hybrid were also mutated (D800L in EGFR T790M and D381L in Bcr-Abl E255K, respectively). Recombinant proteins were then prepared using a baculovirus system and used in cell-free biochemical kinase inhibition assay as described in Yuen *et al.*^17^. Consistent with our prediction, while the kinase inhibition effect of erlotinib on both EGFR T790M and EGFR T790M/D800L are not significantly different, the inhibition of a representative Pt-Erlotinib hybrid (**2a**) on EGFR T790M/D800L was reduced significantly about 4 times than that on EGFR T790M (Table 3; Supplementary Fig. S6). This indicates that an additional site (D800) play an important role in the interaction of **2a** to EGFR T790M, presumably allowing the hybrid to maintain its interaction with the EGFR in the presence of the gatekeeper T790M mutation. Similar observation was also obtained in the case of Pt-Imatinib hybrids (Supplementary Table S1).

### Circumvention of TKI resistance mediated by other mechanism

**Figure 4:**
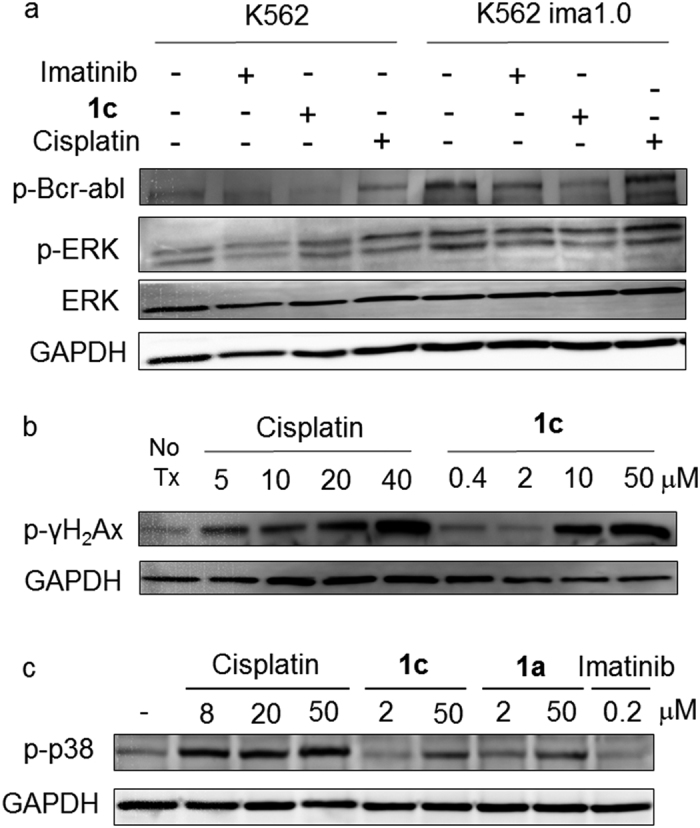
Western blot analysis showing the dual mechanism of action of a representative Pt-imatinib (1a or 1c). (**a**) Phospho-Bcr Abl was upregulated in the imatinib-selected K562 Ima1.0 cells. While imatinib did not appreciably affect phospho-Bcr-Abl, 1c was found to remarkably inhibit phospho-Bcr-Abl at an equipotent concentration. (**b**) A representative Pt-imatinib hybrid (**1c**) was shown to cause a concentration dependent upregulation of the DNA damage marker (phosphor-γ-H2Ax). (**c**) Similar to cisplatin, **1** and **1c** were found to induce p38 phosphorylation whereas imatinib was devoid of this effect.

The circumvention of imatinib resistance by Pt-Imatinib compounds was also observed in another imatinib-selected resistant K562 Ima1.0 subline. In this resistant subline, while no mutation of Bcr-Abl was detected in this resistant subline, the overexpression of Bcr-Abl was found to be the major mechanism contributing to imatinib resistance. Intriguingly, in K562 Ima1.0, a representative Pt-Imatinib hybrid (**1c**) was able to inhibit the activation of the upregulated Bcr-Abl and the downstream ERK1/2 (Fig. 4a), suggesting additional mechanism(s) for circumvention of resistance besides the restoration of drug binding to mutant Bcr-Abl.

To this end, besides the inhibition of Bcr-Abl phosphorylation, the Pt-TKI hybrids were also found to elicit DNA damage, probably through a Pt-mediated pathway where p38MAPK was also found to be activated (Fig. 4b). Interestingly, as expected from the structure of the hybrid compounds, they were found to form only mono Pt-G DNA adduct (Supplementary Fig. S7), in contrast to the classical DNA-Pt diadduct (i.e. crosslink) formed by cisplatin^18^. Therefore, the exact cellular events leading to DNA damage by the hybrids are likely to be different from the classical Pt drugs. Nonetheless, this dual mechanism of action of the hybrid compounds, i.e., inhibition of oncogenic kinase and platination of DNA, is likely also contributing to the circumvention of resistance.

### Hybrid compounds escaped MDR transporters-mediated drug efflux

Most molecular targeted TKIs are substrates of MDR transporters^19^,^20^ whereas Pt drugs are not. The inclusion of Pt in the hybrid structures is therefore hypothesized to bypass P-gp/ABCG2-mediated drug efflux. This could allow the circumvention of transporters-mediated drug resistance and a better penetration of the hybrid compounds across the blood brain barrier for treating brain metastasis.

**Figure 5:**
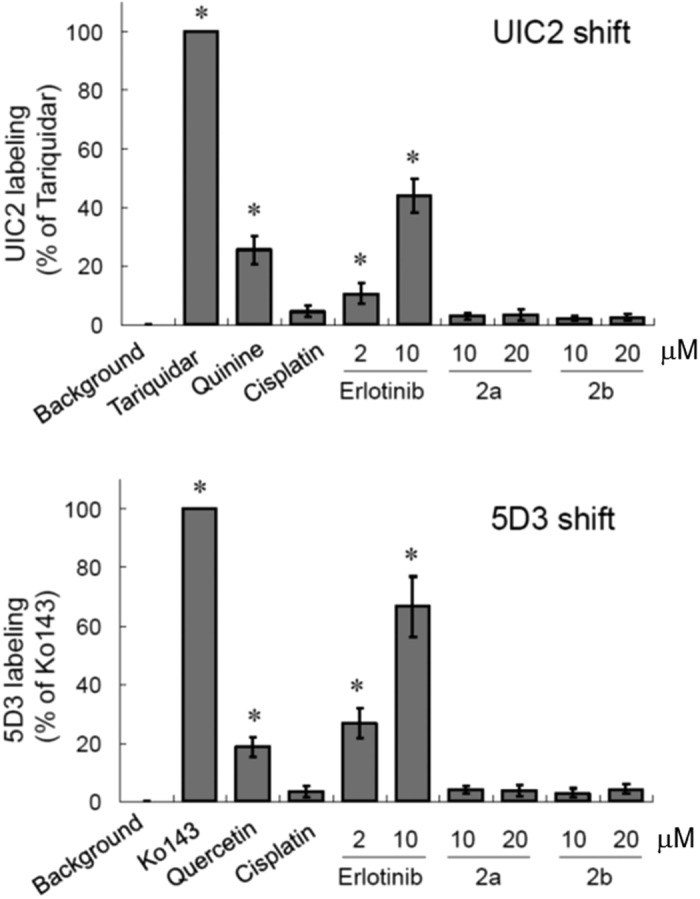
UIC2 and 5D3 shit assay suggesting the lack of interaction between two representative Pt-erlotinib hybrids (2a and 2b) with P-gp (UIC2) and ABCG2 (5D3), respectively. Tariquidar and Ko143 were used as potent P-gp and ABCG2 inhibitor, respectively, to define the 100% antibody shift. Similar to the positive control substrate (quinine: P-gp substrate; quercetin: ABCG2 substrate), the known substrate erlotinib was found to exhibit a concentration dependent antibody shift. Like the non-substrate cisplatin, **2a** and **2b** did not produce any appreciable UIC2 or 5D3 shift.

The lack of interaction between the hybrids and P-gp/ABCG2 was first examined by an antibody-based drug transporter interaction assay (UIC2/5D3 shift assay, respectively). UIC2 and 5D3 are fluorescent and conformation sensitive monoclonal antibody, recognizing an extracellular epitope of P-gp and ABCG2, respectively. The antibody binding to the transporters is known to be increased when the transporter is also bound by its substrate/inhibitor, which can be detected as an increase (right shift) in the fluorescence intensity of the labeled transporter. While erlotinib and imatinib give rise to a concentration-dependent UIC2 and 5D3 shift, all hybrids tested caused remarkably less change in the transporter labeling by the antibodies (Fig. 5). Furthermore, bidirectional transport assay conducted using P-gp or ABCG2-overexpressing MDCKII cells also confirmed that **2a** (one of the Pt-erlotinib hybrids) is not transported (efflux ratio ~1) by both transporters whereas erlotinib is actively pumped across the cell monolayer (efflux ratio about 5 for P-gp and 3 for ABCG2) (Table 4).

An animal study was performed to evaluate the possible enhancement of brain accumulation of Pt-Erlotinib hybrids after a single intravenous dose of the drug (12.5 mg/kg) in BALB/c mice. Mice were sacrificed at 30 min after the intravenous dosing. Brain, liver, kidney were rapidly removed and blood drawn immediately. The drug accumulation in the various organs normalized by the plasma drug concentration were compared between erlotinib, **2a** and cisplatin. As shown in Table 5, the brain accumulation of **2a** was significantly higher (>3 times and 7 times, respectively) than that of erlotinib and cisplatin. Since both P-gp and ABCG2 constitute the blood brain barrier, the enhanced brain penetration of **2a** is likely contributed by the fact that the Pt-Erlotinib hybrids behave as non-substrate of the transporters.

## Discussion

We have demonstrated the use of a hybrid drug design approach, which integrates TKI into Pt coordination compounds, to circumvent resistance mediated by secondary mutation of oncogenic kinases. Interestingly, in the case of erlotinib and vandetanib, the nitrogen atoms on the quinazoline ring cannot be modified without a near complete wipe out of the kinase inhibitory effect^21^. However, we are able to conjugate the TKI to Pt via these nitrogen atoms to make the Pt-erlotinib and Pt-vandetanib hybrids. More importantly, by doing this, we are able to extend additional hydrogen bonds to facilitate the binding of the hybrids to altered sites, so that the hybrids are less affected by the resistance causing secondary mutation. A dual mechanism of anticancer activity of the Pt-TKI hybrids was observed, encompassing inhibition of oncogenic kinases and monofunctional platination of DNA, which also likely contribute to the circumvention of drug resistance. To this end, the formation of mono Pt-G DNA adducts has recently been reported for new monofunctional Pt anticancer agents^22^,^23^. These monofunctional Pt compounds were reported to exhibit new mechanisms of anticancer activity including autophagy^22^ and strong inhibition of transcription^24^. The role of these additional anticancer mechanisms on the apparent circumvention of TKI resistance will need to be investigated.

Apart from being effective in treating tumors bearing both sensitizing and resistance mutations, like the 3^rd^ generation mutant selective EGFR TKI, the hybrids also spare the wild type oncogenic kinases, thus they are also expected to exhibit a better adverse effect profile than the 1^st^ generation TKIs.

This study is also the first to report the application of a hybrid drug design approach to alter the transporter substrate profile of TKIs. Being non-substrates of MDR transporters, the hybrids are not affected by overexpression of the transporters in resistant cells and may be able to penetrate the blood brain barrier to treat cancer metastasis to the brain. We are currently evaluating the *in vivo* properties and pharmacokinetics of these hybrid compounds and conducting an *in-depth* investigation into the apparent dual mechanism of cytotoxic action.

## Methods

### General procedures for the preparation and characterization of the hybrid compounds

The hybrids were synthesized following the general procedure described in Fig. 1 and purified by column chromatography on silica gel (**1a**–**1c**) or size exclusion column (**2a**–**2c**). Mass spectrum was acquired using ESI in positive mode on high resolution FT-ICR mass spectrometer (9.4 Tesla SolariX, Bruker, Billerica, MA, USA). NMR spectra were recorded on Bruker Advance 500 spectrometer. Chemical shift values are expressed in parts per million (ppm) relative to the internal standard tetramethylsilane and coupling constants J are given in Hz. The relative numbers of protons is determined by integration. Specific NMR peaks were assigned by 2D HSQC NMR and coupling experiments (Supplementary Fig. S1b,c,g–i,k). The covalent chemical bond between Pt and the N on pyridine ring of the TKI were unambiguously demonstrated by ^14^N NMR (Supplementary Fig. S1d). The details of structural characterization for representative compound from each series (**1**, **2**, or **3**) can be found in Supplementary Fig. S1a–k.

**1a** (cisplatin-imatinib): pale yellow powder (45% yield). ^1^H NMR (500 MHz, DMSO-d_6_, reference to TMS): δ 2.79 (s, 3 H), 2.22 (s, 3 H), 2.3–2.5 (multiple, 8 H), 3.63 (s, 2 H), 4.25 (s, 6 H, from NH_3_), 7.22 (d, *J* = 8.59 Hz, 1 H), 7.42–7.50 (multiple, 3 H), 7.71 (dd, *J* = 8.25 Hz and 6.00 Hz, 1 H), 7.94 (d, *J* = 7.87 Hz, 2 H), 8.00 (d, *J* = 1.87 Hz, 1 H), 8.57 (d, *J* = 5.25 Hz, 1 H), 8.70 (ddd, *J* = 8.12 Hz, 2.09 Hz, 1.62 Hz, 1 H), 8.84 (dd, *J* = 5.52 Hz, 1.08 Hz, 1 H), 9.17 (s, 1 H), 9.38 (d, *J* = 1.77 Hz, 1 H), 10.17 (s, 1 H). HRMS (m/z): [M]^+^ calculated for C29H37ClN9OPt, 758.2450; found, 758.2439.

**1b** (oxaliplatin-imatinib): pale yellow powder (45% yield). ^1^H NMR (500 MHz, DMSO-d_6_, reference to TMS): δ 1.06 (d, *J* = 13.01 Hz, 10.51 Hz, 2 H), 1.29 (dd, *J* = 17.46 Hz, 14.14 Hz, 2 H), 1.519 (s, 2 H), 1.925 (d, *J* = 15.80 Hz, 12.47 Hz, 2 H), 2.22 (s, 3 H), 2.3–2.5 (multiple, 8 H, 2 H), 2.78 (s, 3 H), 3.65 (s, 2 H), 5.54, 5.68 (ddd, 2 H, from NH_3_), 6.06, 6.31 (dd, 2 H, from NH_3_), 7.22 (d, *J* = 7.93 Hz, 1 H), 7.44 (dd, *J* = 5.14 Hz, 1 H), 7.47 (dd, *J* = 7.51 Hz, 1.98 Hz, 1 H), 7.49 (d, 5.93 Hz, 1 H), 7.75 (dd, *J* = 8.30 Hz and 5.93 Hz, 1 H), 7.95 (d, *J* = 7.91 Hz, 2 H), 7.99 (d, *J* = 2.37 Hz, 1 H), 8.58 (d, *J* = 5.07 Hz, 1 H), 8.72 (ddd, *J* = 8.18 Hz, 1.96 Hz, 1.47 Hz, 1 H), 8.86 (dd, *J* = 5.89 Hz, 0.98 Hz, 1 H), 9.17 (s, 1 H), 9.37 (d, *J* = 1.80 Hz, 1 H), 10.19 (s, 1 H). HRMS (m/z): [M]^+^ calculated for C35H45ClN9OPt, 838.3078; found, 838.3093.

**1c** (transplatin-imatinib): pale yellow powder (45% yield). ^1^H NMR (500 MHz, DMSO-d_6_, reference to TMS): δ 2.79 (s, 3 H), 2.22 (s, 3 H), 2.33, 2.97, 3.03, 3.35 (s, 8 H), 3.68 (s, 2 H), 4.25 (s, 6 H, from NH_3_), 7.22 (d, *J* = 8.21 Hz, 1 H), 7.42 (dd, *J* = 8.10 Hz, 2.03 Hz, 1 H), 7.46 (dd, *J* = 8.51 Hz, 1 H), 7.49 (d, 5.27 Hz, 1 H), 7.73 (dd, *J* = 8.51 Hz and 5.27 Hz, 1 H), 7.95 (d, *J* = 8.51 Hz, 2 H), 8.07 (d, *J* = 1.96 Hz, 1 H), 8.60 (d, *J* = 5.27 Hz, 1 H), 8.69 (ddd, *J* = 8.27 Hz, 3.42 Hz, 1.71 Hz, 1 H), 8.86 (d, *J* = 5.27 Hz, 1 H), 9.13 (s, 1 H), 9.44 (d, *J* = 1.71 Hz, 1 H), 10.23 (s, 1 H). HRMS (m/z): [M]^+^ calculated for C29H37ClN9OPt, 758.2450; found, 758.2455.

**Imatinib** (starting material for **1a**–**1c**, for comparison) ^1^H NMR (500 MHz, DMSO-d_6_, reference to TMS) δ 2.15 (s, 3 H), 2.22 (s, 3 H), 2.2–2.5 (multiple, 8 H), 3.52 (s, 2 H), 7.20 (d, *J* = 8.21 Hz, 1 H), 7.43 (multiple, 3 H), 7.47 (dd, *J* = 8.21 Hz and 1.93 Hz, 1 H), 7.52 (dd, *J* = 7.72 Hz and 4.59 Hz, 1 H), 7.90 (d, *J* = 7.84 Hz, 2 H), 8.08 (d, *J* = 1.96 Hz, 1 H), 8.47 (ddd, *J* = 8.23 Hz, 3.50 Hz, 2.06 Hz, 1 H), 8.51 (d, *J* = 5.35 Hz, 1 H), 8.68 (dd, *J* = 4.94 Hz and 1.85 Hz, 1 H), 8.96 (s, 1 H), 9.27 (d, *J* = 2.17 Hz, 1 H), 10.16 (s, 1 H).

**2a** (cisplatin-erlotinib): pale yellow powder (25% yield). ^1^H-NMR (DMSO-d_6_, reference to TMS), δ (ppm): 10.18 (br, 1 H), 8.73 (s, 1 H), 8.64 (s, 1 H), 7.93 (s, 1 H), 7.85 (m, 1 H), 7.75 (d, 1 H), 7.48 (t, 1 H), 7.34 (s, 1 H), 4.53–4.54 (m, 1 H), 4.44 (br, 2 H), 4.32–4.34 (br, 7 H), 4.26 (s, 1 H), 3.80–3.86 (m, 4 H), 3.40 (s, 3 H), 3.37 (s, 3 H). HRMS (m/z): [M]^+^ calculated for C_22_H_29_ClN_5_O_4_Pt, 658.1546; found, 658.1550. Purity (HPLC, λ = 247 nm): 98.2%.

**2b** (oxaliplatin-erlotinib): pale yellow powder (28% yield). ^1^H-NMR (DMSO-d_6_, reference to TMS), δ (ppm): 10.19 (s, 1 H), 8.75 (s, 1 H), 8.61 (s, 1 H), 7.95 (s, 1 H), 7.86 (s, 1 H), 7.75 (d, 1 H), 7.48 (t, 1 H), 7.35 (d, 1 H), 6.02 (br, 2 H), 5.55 (br, 1 H), 5.35 (br, 1 H), 4.34 (t, 4 H), 4.26 (s, 1 H), 3.79–3.87 (m, 4 H), 3.40 (s, 3 H), 3.37 (s, 3 H), 2.20–2.34 (m, 2 H), 1.94 (d, 1 H), 1.87 (d, 1 H), 1.51 (m, 2 H), 1.32 (m, 2 H), 1.06 (t, 2 H). MS (m/z): [M]^+^ calculated for C28H37ClN5O4Pt, 738.5; found, 738.5. Purity (HPLC, λ = 247 nm): 91.5%.

**2c** (transplatin-erlotinib): pale yellow powder (42% yield). ^1^H-NMR (DMSO-d_6_, reference to TMS), δ (ppm): 10.16 (s, 1 H), 8.82 (s, 1 H), 8.55 (s, 1 H), 7.93 (s, 1 H), 7.85 (s, 1 H), 7.75 (d, 1 H), 7.49 (t, 1 H), 7.34 (d, 1 H), 4.55 (t, 2 H), 4.34 (t, 2 H), 4.27 (s, 1 H), 4.06 (br, 6 H), 3.87 (t, 2 H), 3.79 (t, 2 H), 3.42 (s, 3 H), 3.37 (s, 3 H). MS (m/z): [M]+ calculated for C22H29ClN5O4Pt 658.5; found, 658.4. Purity (HPLC, λ = 247 nm): 94.9%.

**Erlotinib** (starting material for **2a**–**2c**, for comparison: ^1^H-NMR (500 MHz, DMSO-d_6_, reference to TMS). δ (ppm): 9.48 (s, 1 H), 8.50 (s, 1 H), 7.99 (t, 1 H), 7.90 (d, 1 H), 7.87 (s, 1 H), 7.41 (d, 1 H), 7.23 (s, 1 H), 7.20 (d, 1 H), 4.28–4.31 (m, 4 H), 4.20 (s, 1 H), 3.74–7.80 (m, 4 H), 3.35 (s, 6 H).

**3a** (cisplatin-vandetanib): pale grey powder (67.7% yield). ^1^H NMR (DMSO-d_6_, reference to TMS), δ (ppm): 1.4513 (tdd, J = 12.3 Hz, 3.19 Hz, 2 H), 1.8716 (dd, J = 12.90 Hz, 2 H), 1.9009–2.0619 (m, 3 H), 2.2416 (s, 3 H), 2.8933 (dd, J = 11.12 Hz, 2 H), 4.0175 (s, 3 H), 4.0911 (ds, J = 7.42 Hz, 1 H), 4.2579 (dd, J = 7.82 Hz, 1 H), 4.3477 (s, 3 H), 4.4796 (s, 3 H), 7.5011 (dd, J = 8.36 Hz, 1 H), 7.5657 (dd, J = 9.88 Hz, 1.98 Hz, 8.54 Hz, 1 H), 7.7720 (dd, J = 9.86 Hz, 1.99 Hz, 1 H), 7.8968 (s, 1 H), 8.5839 (s, 1 H), 8.6417 (s, 1 H). HRMS (m/z): [M]^+^ calculated for C22H30BrClFN6O2Pt, 739.0909; found, 739.0901. Purity (HPLC, λ = 255 nm): 98.1%.

**3b** (oxaliplatin-vandetanib): pale grey powder (77.9% yield). ^1^H NMR (DMSO-d_6_, reference to TMS), δ (ppm): ^1^H NMR: δ 1.0447 (t, J = 10.95 Hz, 3 H), 1.1805–1.3772 (m, 2 H), 1.4349 (dd, J = 12.39, 2 H), 1.0510 (dd, J = 7.73, Hz, 2 H), 2.0950–1.8910 (m, 5 H), 2.2414 (s, 3 H), 2.2570 (m, 2 H), 2.8962 (dd, J = 9.36 Hz, 2 H), 3.9881 (s, 3 H), 4.0009–4.3600 (m, 2 H), 5.3630 (td, J = 9.84 Hz, 1 H), 5.5105 (td, J = 9.83 Hz, 1 H), 6.0316 (d, J = 7.53 Hz, 2 H), 7.4740 (dd, J = 8.40 Hz, 1 H), 7.5372 (dd, J = 8.44 Hz, 1.72 Hz, 1 H), 7.7433 (dd, J = 9.88 Hz, 1.98 Hz, 1 H), 7.8787 (s, 1 H), 8.5408 (s, 1 H), 8.6352 (s, 1 H). HRMS (m/z): [M]^+^ calculated for C28H38BrClFN6O2Pt, 819.1537; found, 819.1571. Purity (HPLC, λ = 255 nm): 84.71%.

**3c** (transplatin-vandetanib): pale grey powder (71.5% yield). ^1^H NMR (DMSO-d_6_, reference to TMS), δ (ppm): ^1^H NMR: δ 1.4603 (tdd, J = 12.45 Hz, 2.73 Hz, 2 H), 1.8700 (dd, J = 12.54 Hz, 2 H), 1.9116 (m, 1 H), 2.0620 (m, 2 H), 2.2665 (s, 3 H), 2.9240 (dd, J = 8.92 Hz, 2 H), 3.9809 (s, 3 H), 4.0507 (s, 6 H), 4.2423 (d, J = 5.83 Hz, 2 H), 7.4351 (dd, J = 8.31 Hz, 1 H), 7.5380 (dd, J = 8.30 Hz, 1.54 Hz, 1 H), 7.7503 (dd, J = 9.77 Hz, 2.01 Hz, 1 H), 7.8623 (s, 1 H), 8.4429 (s, 1 H), 8.7069 (s, 1 H). HRMS (m/z): [M]^+^ calculated for C22H30BrClFN6O2Pt, 739.0909; found, 739.0901. Purity (HPLC, λ = 255 nm): 98.5%.

**Vandetanib** (starting material for **3a**–**3c**; for comparison). ^1^H NMR (DMSO-d_6_, reference to TMS), δ (ppm): ^1^H NMR: δ 1.3518 (tdd, J = 12.9 Hz, 2.7 Hz, 2 H), 1.7634 (dd, J = 10.63 Hz, 2 H), 1.7724 (m, 1 H), 1.8728 (dd, J = 10.74 Hz, 2 H), 2.1612 (s, 3 H), 2.7903 (dd, J = 11.26 Hz, 2 H), 3.9437 (s, 3 H), 4.0009 (d, J = 5.95 Hz, 2 H), 7.1778 (s, 1 H), 7.4643 (dd, J = 8.54 Hz, 1.92 Hz, 1 H), 7.5304 (dd, J = 8.12 Hz, 8.54 Hz, 1 H), 7.6566 (dd, J = 10.04 Hz, 2.12 Hz, 1 H), 7.7893 (s, 1 H), 9.5255 (s, 1 H).

### Cell culture

A panel of NSCLC cell lines harboring different EGFR mutations were purchased from American Type Culture Collection (ATCC; Manassas, VA, USA). They include H292 (bearing wild type EGFR), HCC827 (bearing EGFR sensitizing E746_A750 deletion) and H1975 (bearing the resistance causing EGFR T790M secondary mutation). The leukemic cell line K562 (harboring wild type Bcr Abl) and RPMI8226 (no Bcr Abl expression) were generous gift from Dr. Susan Bates (National Cancer Institute, NIH, Bethesda, MD, USA). An imatinib-selected resistant subline K562 Ima1.0 was derived from the parental K562 cells by incubating in progressively elevated concentration of imatinib up to 1 μM. At the time of experiments, it was about 15-fold resistant to imatinib. The HEK293T cells (from ATCC) were stably transfected with the wild type Bcr Abl or its E255K mutant. H292, HCC827, H1975, RPMI8226 cells were maintained in RPMI1640 medium supplemented with 10% fetal bovine serum, 100 units/mL streptomycin sulfate, and 100 units/mL penicillin G sulfate, and incubated at 37 °C in 5% CO_2_. K562, K562 Ima1.0, HEK293T stable transfected cell lines were grown in DMEM medium supplemented with 10% fetal bovine serum, 100 units/mL streptomycin sulfate, 100 units/mL penicillin G sulfate.

### Cell-free biochemical kinase inhibition assay

Inhibition of Tyr-kinase signaling by the Pt-TKI hybrids was examined in a cell-free system by assessing the phosphorylation of a poly-EY (for EGFR) or abltide (for Bcr-ABl) substrate with recombinant EGFR^wt^, EGFR^L858R^, EGFR^L858R+T790M^, Bcr-Abl^wt^ or Bcr-Abl^E255K^ proteins, respectively. Kinase inhibition by the tested hybrid compounds was evaluated by using the ADP-Glo Kinase assay kit (Promega, Madison, WI, USA).

To specifically demonstrate the binding of Pt-erlotinib/Pt-imatinib hybrids via additional hydrogen bonds than the original TKI to the kinase domain of EGFR/Bcr-Abl respectively, we created EGFR T790M (purchased from GeneCopoeia (Rockville, MD, USA))/Bcr Abl constructs (obtained from Addgene (Cambridge, MA, USA)) in which a predicted additional interaction site by the hybrid was also mutated (D800L in EGFR T790M and D381L in Bcr-Abl). Site directed mutagenesis was performed with the GeneArt Site-Directed Mutagenesis System according to manufacturer’s instruction (Life Technologies, Grand Island, NY, USA). Recombinant proteins were then prepared using a baculovirus expression system (BacPak, Sf21 cells; Clontech, Mountain View, CA, USA) according to the manufacturer’s protocol and used in cell-free biochemical kinase inhibition assay as described in Yuen *et al.*^17^. The cell-free kinase inhibition assay was performed as described above.

### Docking simulation analysis

AutoDock 4 (version 4.2.6, The Scripps Research Institute, La Jolla, CA, USA) was used for the docking studies. Our docking simulation method was first validated by comparing the docking result from known pairs of interacting drug-kinase (i.e., erlotinib-EGFR and imatinib-Bcr Abl) with the actual binding data from the reported crystal structure (pdb: 1M17, wild-type EGFR-erlotinib^25^; 1IEP, Bcr Abl-imatinib^26^). All Pt-Erl hybrids were optimized in water as solvent box by Gaussian 09 using B3LYP method with mixed basis set 6–31 + g* for atoms C, O, N, H and Cl and LANL2DZ for Pt. Prediction of binding between Pt-Erlotinib hybrids and EGFR (pdb: wild type–1M17; sensitizing mutation (L858R)–4I20; secondary and resistance causing mutation (L858R/T790M)–4I22), and between Pt-Imatinib hybrids and Bcr-Abl (pdb: wild type–1IEP; Bcr Abl–1IEP artificially changed to E255K by the AMBER software (AMBER14, University of California, San Franciso). Docking simulation was performed by Autodock4 using Lamarckian GA algorithm. The van der Waals radius and well of depth of Pt were adopted from a previously published paper for docking studies involving Pt atom^27^. Briefly, Kollman charge and Gasteiger charge were added to protein and ligand respectively. Blind docking was performed to search for the binding site, which was then followed by 2^nd^ docking study for the accurate binding conformation of structure. The structures were visualized by VMD (version 1.9.1)^28^ or GaussView 5.0.

### Western blot analysis

NSCLC (HCC827, H1975) or CML (K562) cell lines were treated with the tested hybrids for the designated time (2 or 24 h). The cells were then harvested for Western blot analysis in lysis buffer (0.05M HEPES pH7.4, 0.15M NaCl, 10% v/v glycerol, 1% v/v Triton X-100, 2 mM EDTA) supplemented with protease and phosphatase inhibitor cocktail (Thermo Scientific). Whole cell lysates were separated by SDS-PAGE and subjected to immunoblot analysis with the respective antibodies (phosphor-p38, phosphor-Bcr-abl (Tyr-177) (Cell Signaling Technology, Danvers, MA, USA); total EGFR, phosphor-EGFR (Y845), PARP, phosphor-ERK1/2 (Thr177/Thr160), ERK1/2, phosphor-Akt, total Akt, phosphor-histone H2Ax (Ser139), phosphor-ATM, and GAPDH (Santa Cruz Biotechnology, Santa Cruz, CA, USA). Primary antibody incubation was carried out at 4 °C overnight in 5% BSA/PBS-T. Afterwards, the membranes were incubated with HRP-conjugated donkey anti-mouse/anti-rabbit secondary antibody at room temperature for 1 h, and developed using the WesternBright Quantum chemiluminescence detection system (Advansta Corporation, Menlo Park, CA). Anti-GAPDH antibody was used as the loading control (Santa Cruz Biotech, Santa Cruz, CA). Digital chemiluminescence images were captured and analyzed by using the FluorChem Q Imaging System (Alpha Innotech Corporation, Santa Clara, CA, USA).

### UIC2 and 5D3 labeling assay

The binding of the conformational sensitive UIC2 and 5D3 antibody to intact cells (P-gp overexpressing SW620 Ad300 or ABCG2-overexpressing S1M1-80) in the presence or absence of the tested hybrids was measured by flow cytometry as described previously^29^. Briefly, cells were pre-incubated with the tested compounds in 0.5% bovine serum albumin/Dulbecco’s PBS for 10 min at 37 °C before labeling with 0.5 μg/mL of PE-conjugated anti-Pgp UIC2, PE-conjugated anti-ABCG2 antibody 5D3 or PE-conjugated mouse IgG2b negative control antibody for another 45 min at 37 °C. As positive control for maximum labeling, UIC2 and 5D3 binding was determined in the presence of 1 μM tariquidar (specific P-gp inhibitor) and 1 μM Ko143 (specific ABCG2 inhibitor), respectively.

### Bidirectional drug transport assay

MDCKII-Pgp or MDCKII-ABCG2 cells were seeded onto Transwell Permeable support (Costar Corning; pore size of 0.4 μm and a surface area of 1.12 cm^2^ in 12-well plate). The medium was changed every other day until the 5th day after seeding. The transepithelial electrical resistance (TEER) values of cell monolayers were measured periodically. On the 5^th^ day, only those monolayers with a TEER values greater than 400 Ωcm^2^ were employed for the transport study to ensure the integrity of the monolayers formed on the filters. PBS supplemented with Ca^2+^ and Mg^2+^ at pH 7.4 was prepared and used as the transport buffer. The transport study was initiated by loading the tested compounds at 10 μM into the “donor” chamber (i.e., the apical side of the cell monolayer with 0.5 mL of transport buffer). Then aliquots of 0.5 mL samples were taken from the “receiver” side at different time points (15, 30, 45, 60, 90 and 120 min). The removed volume was replaced with a pre-warmed blank PBS. The transport activities were measured in both directions apical to basolateral (A-B) and basolateral to apical side (B-A). Sample from triplicate transwells were collected and stored at −20 °C until analysis by LC-MS or ICP-OES. Apparent permeability (Papp) and efflux ratio were calculated according to the following formula:


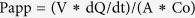



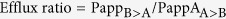


where dQ/dt represents the change of drug concentration in the receiver chamber during the time, V is the volume of the solution in the receiver chamber and A is the membrane surface area, Co is the loading concentration in the donor chamber.

### Assessment of drug accumulation in the brain of BALB/c mice

The accumulation of the tested compounds (cisplatin, erlotinib and **2a**) in various organs (brain, liver, kidney) was evaluated in BALB/c mice (n = 6) after intravenous injection. Briefly, the tested compounds were administered intravenously at the concentration of 12.5 mg/kg. The mice were sacrificed after 30 min of drug administration followed immediately by the collection of brain, kidney, liver and plasma. PBS was added to the tissues and homogenized. 150 μL of the tissue lysate and 50 μL of the plasma sample were transferred to centrifuge tube and stored at −80 °C until further treatment. For samples obtained from erlotinib-treated mice, 500 μL dimethylformamide and 50 μL of internal standard gefitinib solution were added in each sample and shaken vigorously for 2 min. The samples were centrifuged at 14,000 rpm for 5 min at 4 °C and 500 μL supernatant was withdrawn and dried under vacuum. The samples were reconstituted with 200 μL mobile phase (1:1 of acetonitrile/5 mM ammonium formate, pH 4.31) and centrifuged at 14,000 rpm at 4 °C for 5 min. 150 μL of supernatant were transferred for HPLC/MS/MS analysis. For samples obtained from cisplatin or **2a**-treated mice, the tissue lysate were digested with concentrated HNO_3_ (500 μL) at 80 °C until no tissue debris observed. The digested lysate was diluted with 1% HNO_3_ and analyzed by ICP-OES. The ratio of “brain drug accumulation to plasma drug concentration” was compared among the tested compounds.

## Additional Information

**How to cite this article**: Wei, Y. *et al.* A platinum-based hybrid drug design approach to circumvent acquired resistance to molecular targeted tyrosine kinase inhibitors. *Sci. Rep.* **6**, 25363; doi: 10.1038/srep25363 (2016).

## Supplementary Material

Supplementary Information

## Figures and Tables

**Table 1:** Anticancer activity (IC_50_, μM) of the hybrid compounds in cell lines harboring oncogenic kinases with different mutations.

	NSCLC			CML cell line	
	H292	HCC827	H1975*	K562	RPMI8226
EGFR status	Wild type	ΔE746-A750 (sensitizing)	L858R & T790M (resistant)	NA	NA
Bcr-Abl status	NA	NA	NA	Bcr-Abl positive	Bcr-Abl negative
Erlotinib	0.22 ± 0.12	0.0010 ± 0.0005	11.83 ± 3.19 (11830)	ND	ND
Pt-Erlotinib **(2a)**	3.27 ± 1.75	0.16 ± 0.03	29.58 ± 3.07 (185)	ND	ND
Pt-Erlotinib **(2b)**	1.33 ± 0.39	0.12 ± 0.07	18.18 ± 3.68 (152)	ND	ND
Pt-Erlotinib **(2c)**	0.33 ± 0.02	0.006 ± 0.002	3.17 ± 2.25 (528)	ND	ND
Imatinib	ND	ND	ND	0.15 ± 0.02	11.83 ± 0.91
Pt-Imatinib **(1a)**	ND	ND	ND	1.86 ± 0.29	95.42 ± 5.79
Pt-Imatinib **(1b)**	ND	ND	ND	2.27 ± 0.33	94.02 ± 3.71
Pt-Imatinib **(1c)**	ND	ND	ND	1.04 ± 0.01	46.95 ± 3.99
Vandetanib	6.24 ± 0.94	0.011 ± 0.002	13.84 ± 2.06 (1258)	ND	ND
Pt-Vandetanib **(3a)**	26.45 ± 3.19	0.42 ± 0.08	28.30 ± 3.18 (67)	ND	ND
Pt-Vandetanib **(3b)**	9.32 ± 1.01	0.47 ± 0.01	8.28 ± 0.58 (18)	ND	ND
Pt-Vandetanib **(3c)**	18.45 ± 4.48	0.043 ± 0.007	21.25 ± 1.09 (494)	ND	ND
Cisplatin	2.12 ± 0.87	6.65 ± 1.66	7.50 ± 2.04 (1.1)	19.76 ± 1.42	2.97 ± 0.82
Oxaliplatin	3.77 ± 0.35	3.60 ± 1.76	5.17 ± 2.34 (1.4)	16.55 ± 2.08	0.43 ± 0.04

NA = not applicable; ND = not determined. *Fold resistance (IC_50_ in H1975/IC_50_ in HCC827) is indicated in parenthesis.

**Table 2:** Anticancer activity (IC_50_, μM) of Pt-imatinib hybrids in HEK293 cells stably transfected with wild type (WT) Bcrl-Abl or Bcr-Abl E255K mutant, and an imatinib-selected drug resistant K562 subline (K562 Ima1.0).

	HEK293			K562	
	Mock transfected	Bcr-Abl wild type	Bcr-Abl E255K*	Sensitive parental	Imatinib selected (K562 Ima1.0)*
Genetic abormalities	Bcr-Abl negative	Bcr-Abl wild type	Bcr-Abl E255K	Nil	Bcr-Abl amplification
Imatinib	11.58 ± 2.19	0.69 ± 0.11	10.68 ± 1.15 (15)	0.15 ± 0.02	2.08 ± 0.48 (14)
Pt-Imatinib **(1a)**	12.67 ± 1.47	1.55 ± 0.32	1.96 ± 0.43 (1.3)	1.86 ± 0.29	3.41 ± 0.46 (1.8)
Pt-Imatinib **(1b)**	10.56 ± 1.58	1.44 ± 0.25	2.01 ± 0.55 (1.4)	2.27 ± 0.33	2.92 ± 0.93 (1.3)
Pt-Imatinib **(1c)**	10.13 ± 1.39	1.13 ± 0.41	1.21 ± 0.57 (1.1)	1.04 ± 0.01	1.05 ± 0.14 (0.9)
Cisplatin	5.43 ± 0.72	8.16 ± 1.01	6.54 ± 0.49 (0.8)	19.76 ± 1.42	17.47 ± 1.06 (0.9)
Oxaliplatin	2.41 ± 0.32	3.15 ± 0.87	2.65 ± 0.71 (0.8)	16.55 ± 2.08	16.43 ± 1.14 (1.0)

^*^Fold resistance is indicated in parenthesis.

**Table 3:** Inhibitory effect of erlotinib, imatinib, Pt-erlotinib and Pt-imatinib hybrids on the cell-free *in vitro* kinase assay of wild type and point mutated EGFR and Bcr-Abl kinase.

	C_50_ (nM)					
	Imatinib	1a	1b	Erlotinib	2a	2b
Bcr Abl kinase	(fold over wild type)					
Wild type	27.45 ± 0.41	165.2 ± 51.8	106.8 ± 4.4	NA	NA	NA
E255K	>10000 (>364)	842.1 ± 50.5 (5.1)	1123.3 ± 150.7 (10.5)	NA	NA	NA
E255K/D381L	>10000 (>364)	2343.0 ± 563.3 (14.2)*	4109.0 ± 343.1 (38.5)*	NA	NA	NA
EGFR kinase				(fold over L858R)		
Wild type	NA	NA	NA	0.84 ± 0.15 (70)	20.58 ± 1.71 (16.9)	3.76 ± 0.48 (7.4)
L858R	NA	NA	NA	0.012 ± 0.001	1.22 ± 0.18	0.51 ± 0.08
L858R/T790M	NA	NA	NA	220.7 ± 15.0 (18392)	863.0 ± 191.5 (707)	333.7 ± 25.2 (654)
L858R/T790M/D800L	NA	NA	NA	264.5 ± 13.2 (22042)	3301.0 ± 290.7 (2706)^#^	1765.7 ± 194.2 (3462)^#^

*p < 0.05, compared with inhibition on E255K; ^#^p < 0.05, compared with inhibition on L858R/T790M; NA = not applicable.

**Table 4:** Bidirectional drug transport assay showing that erlotinib are effectively pumped by P-gp and ABCG2 from the basolateral side to the apical side whereas the new hybrids are not.

	MDCKII–P-gp			MDCKII–ABCG2		
	A to B	B to A	Efflux Ratio	A to B	B to A	Efflux Ratio
Erlotinib	1.84 × 10^−5^	7.42 × 10^−5^	4.56 ± 0.15	1.49 × 10^−5^	4.54 × 10^−5^	3.09 ± 0.40
Cisplatin	5.81 × 10^−6^	6.05 × 10^−6^	1.04 ± 0.09*	6.47 × 10^−6^	3.09 × 10^−6^	0.52 ± 0.20*
**2a**	3.32 × 10^−6^	2.82 × 10^−6^	1.02 ± 0.11*	7.21 × 10^−6^	7.88 × 10^−6^	1.09 ± 0.26*
**2b**	6.59 × 10^−6^	6.48 × 10^−6^	1.02 ± 0.12*	ND	ND	ND
**2c**	3.57 × 10^−6^	3.40 × 10^−6^	0.97 ± 0.09*	ND	ND	ND

The mean value from three independent experiments was shown for the A to B and B to A transport. ND = not determined. (*p < 0.05, difference from erlotinib).

**Table 5:** Relative accumulation of drug in various organs after an intravenous dose (12.5 mg/kg) of erlotinib, cisplatin and 2a in BALB-c mice (n = 6).

	Drug organ accumulation/plasma drug concentration		
	Brain	Kidney	Liver
Erlotinib	8.93 × 10^−4^	1.35 × 10^−2^	4.36 × 10^−2^
	±4.34 × 10^−4^	±0.81 × 10^−2^	±1.80 × 10^−2^
Cisplatin	4.14 × 10^−4^	2.26 × 10^−2^	1.13 × 10^−2^
	±2.70 × 10^−4^	±1.27 × 10^−2^	±0.70 × 10^−2 ^*
2a	3.12 × 10^−3^	3.05 × 10^−2^	6.44 × 10^−2^
	±3.45 × 10^−3^ *	±1.83 × 10^−2^	±5.24 × 10^−2^

(*p < 0.05, difference from erlotinib).
