# Supplementary material for: A platinum-based hybrid drug design approach to circumvent acquired resistance to molecular targeted tyrosine kinase inhibitors
Source: Sci Rep. 2016 May 6;6:25363. doi: 10.1038/srep25363 (PMC4858680; doi:10.1038/srep25363)
Supplement: Supplementary Information [file srep25363-s1.pdf]

A platinum-based hybrid drug design approach to circumvent acquired resistance to molecular targeted tyrosine kinase inhibitors

Yuming Wei,[1, ‡] Daniel C. Poon,[2, ‡] Rong Fei,[1, ‡] Amy S.M. Lam,[2] Steve C.F. Au-Yeung,[1,*] Kenneth K.W.To[2,*]

[1] Department of Chemistry, Faculty of Science, The Chinese University of Hong Kong, Hong Kong SAR, China
[2] School of Pharmacy, Faculty of Medicine, The Chinese University of Hong Kong, Hong Kong SAR, China

Supplementary Table S1. Comparison of the inhibition of the Pt-TKI hybrids (versus the original TKI) on a panel of 132 oncogenic kinases.

**Percent control**	Imatinib	**1b**		Erlotinib	**2a**		Vandetanib	**3b**
ABL1(E255K)-phosphorylated	1.7	9.6		0.6	9.9		0.1	0.7
ABL1(E317I)-nonphosphorylated	0.1	0		45.1	83		7.1	22
ABL1(F317I)-phosphorylated	5.5	7.8		9.9	49		1.7	9.2
ABL1(F317L)-nonphosphorylated	0.0	0		6.0	61		0.7	12
ABL1(F317L)-phosphorylated	1.1	10		1.5	25		0.3	5.4
ABL1(H396P)-nonphosphorylated	0.1	0.1		0.9	0.9		0.1	0.2
ABL1(H396P)-phosphorylated	0.6	8.3		0.6	13		0.1	0.2
ABL1(M351T)-phosphorylated	0.4	8.3		0.7	15		0.1	1.0
ABL1(Q252H)-nonphosphorylated	0.0	1.6		2.2	13		0.2	0.2
ABL1(Q252H)-phosphorylated	0.9	7		0.6	12		0.2	0.5
ABL1(T315I)-nonphosphorylated	100	91		5.8	41		1.5	0.3
ABL1(T315I)-phosphorylated	100	82		0.6	30		0.2	1.9
ABL1(Y253F)-phosphorylated	1.3	22		0.8	16		0.1	0.4
ABL1-nonphosphorylated	0.0	0		3.2	25		0.5	1.2
ABL1-phosphorylated	0.2	3.6		0.8	16		0.2	0.4
ABL2	0.1	11		2.0	46		0.7	20
ALK	100	99		10.7	79		17.4	29
AXL	100	100		28.6	93		2.4	19
BLK	4.9	12		1.9	41		0.7	1.4
BMX	100	98		100	100		100	92
BRK	100	93		100	89		1.6	27
BTK	100	100		100	87		14.5	37
CSF1R	0.1	0.5		100	91		10.7	42
CSK	100	100		49.0	100		20	41
DDR1	0.0	0.2		7.3	74		0.1	0.3
DDR2	0.1	10		100	100		3.1	0.21
EGFR	100	89		0.0	0		0.1	2.1
EGFR(E746-A750del)	100	75		0.0	0.2		0.1	3.2
EGFR(G719C)	100	72		0.0	0.6		0.1	0.5
EGFR(G719S)	100	79		0.0	0.1		0.1	0.9
EGFR(L747-E749del,A750P)	43.2	67		0.0	0.1		0.1	0.4
EGFR(L747-S752del,P753S)	100	81		0.0	0		0.1	3.8
EGFR(L747-T751del,Sins)	100	76		0.0	2.2		0.1	1.6
EGFR(L858R)	100	81		0.0	0		0.1	0.8

EGFR(L858R,T790M)	100	100		1.9	8.8		2.2	4.8
EGFR(L861Q)	100	42		0.0	0.1		0.1	0.3
EGFR(S752-I759del)	100	46		0.0	9.8		0.1	2.2
EGFR(T790M)	100	78		1.4	15		1.0	7.4
EPHA1	100	86		100	73		2.2	11
EPHA2	100	99		100	93		9.9	43
EPHA3	100	64		19.4	60		16.7	30
EPHA4	100	88		100	79		13.8	42
EPHA5	100	100		6.6	100		2.3	78
EPHA6	100	90		4.2	71		0.5	6.3
EPHA7	100	100		12.3	67		19.4	79
EPHA8	12.3	68		8.6	56		0.9	6.8
EPHB1	100	94		9.9	90		2.8	31
EPHB2	100	95		100	94		4.2	69
EPHB3	100	87		100	88		100	100
EPHB4	100	100		18.0	91		4.9	35
EPHB6	100	92		14.5	76		0.8	2.5
ERBB2	100	82		22.5	21		20.6	16
ERBB3	100	100		9.9	100		1.6	4.1
ERBB4	100	93		2.2	47		4.5	56
FAK	100	90		100	94		100	86
FER	100	89		100	95		100	90
FES	100	96		100	98		100	100
FGFR1	100	96		100	77		5.3	40
FGFR2	100	100		100	91		9.9	67
FGFR3	100	94		100	89		13.8	62
FGFR3(G697C)	100	82		100	81		40.8	95
FGFR4	100	94		100	99		18.7	97
FGR	19.4	80		9.9	81		2.6	33
FLT1	100	85		30.6	88		2.5	21
FLT3	100	88		10.7	75		7.8	49
FLT3(D835H)	100	88		3.4	51		5.3	8.8
FLT3(D835Y)	100	81		1.3	21		7.7	46
FLT3(ITD)	38.7	74		7.6	79		15.3	62
FLT3(K663D)	100	84		11.5	67		1.9	43
FLT3(N841I)	100	72		4.8	70		10.7	18
FLT3(R834Q)	100	94		100	80		11.5	89

FLT4	100	99		17.4	90		9.9	14
FRK	13.0	58		16.7	75		1.7	56
FYN	23.7	78		100	72		3.5	32
HCK	100	82		15.3	64		3.5	3.2
IGF1R	100	100		100	86		100	100
INSR	100	92		100	85		100	72
INSRR	100	100		100	100		100	100
ITK	100	100		100	84		100	95
JAK1(JH1domain-catalytic)	100	80		100	93		100	83
JAK1(JH2domain-pseudokinase)	100	100		100	100		100	85
JAK2(JH1domain-catalytic)	100	100		27.0	46		100	100
JAK3(JH1domain-catalytic)	100	55		6.5	28		100	100
KIT	0.1	0.2		14.5	86		2.5	1.1
KIT(A829P)	0.1	5		100	56		0.3	2.7
KIT(D816H)	5.3	41		100	70		4.0	30
KIT(D816V)	8.9	51		13.8	90		2.8	12
KIT(L576P)	0.1	0.9		25.9	73		1.4	2.2
KIT(V559D)	0.1	0		23.7	88		1.8	0.5
KIT(V559D,T670I)	20	57		11.5	71		16.7	85
KIT(V559D,V654A)	0.9	3.1		100	100		5.3	19
LCK	30	11		2.4	38		0.2	0.7
LTK	92	85		8.2	82		5.2	27
LYN	74	67		5.0	69		1.1	21
MERTK	100	100		8.9	100		12.3	17
MET	100	88		27.5	63		36.3	80
MET(M1250T)	100	89		21.9	95		100	87
MET(Y1235D)	100	100		9.9	92		29.1	52
MST1R	100	100		100	100		100	100
MUSK	100	100		100	97		100	95
PDGFRA	0.3	1.2		15.3	100		2.2	5.7
PDGFRB	0.1	0.1		12.3	85		0.9	1.6
PYK2	100	92		100	90		100	80
RET	100	95		11.5	46		0.3	0.25
RET(M918T)	100	88		3.2	61		0.1	0.2
RET(V804TL)	100	100		100	100		100	94
RET(V804M)	100	82		100	71		100	100
ROS1	100	32		100	91		100	74

SRC	100	92		6.5	66		0.7	0.5
SRMS	100	98		100	94		16.0	95
SYK	100	93		100	88		100	85
TEC	100	100		100	72		100	100
TIE1	100	100		7.8	77		13.0	60
TIE2	100	94		100	94		9.1	49
TNK1	100	90		5.9	85		100	90
TNK2	100	100		100	100		100	100
TRKA	100	98		100	94		100	92
TRKB	100	100		100	95		100	72
TRKC	100	100		100	93		100	98
TXK	100	87		27.5	76		27.0	56
TYK2(JH1domain-catalytic)	100	94		100	92		100	83
TYK2(JHSdomain-pseudokinase)	46.5	100		19.4	100		100	100
TYRO3	100	80		28.1	85		0.9	0
VEGFR2	100	100		36.3	98		7.6	3.2
YES	100	100		18.0	100		1.2	23
ZAP70	100	100		100	89		100	99

Supplementary Table S2. Predicted interacting amino acid residues and length of hydrogen bond formed (Å) between Pt-TKI hybrids and the corresponding oncogenic kinase. NA = not applicable.

	**Predicted hydrogen bond length (Å)**							
	**Imatinib**	**1a**	**1b**	**1c**	**Erlotinib**	**2a**	**2b**	**2c**
**Bcr-Abl E255K**								
**Glu286**	2.02	--	2.24	--	NA	NA	NA	NA
**Thr315**	2.40	--	--	--	NA	NA	NA	NA
**Met318**	1.99	--	--	--	NA	NA	NA	NA
**Asp381**	1.83	1.68	1.81	1.93	NA	NA	NA	NA
**His361**	--	2.31	1.98	2.03	NA	NA	NA	NA
**Arg362**	--	--	--	2.07	NA	NA	NA	NA
**Asp363**	--	1.75	--	--	NA	NA	NA	NA
**EGFR L858R/T790M**								
**Lys716**	NA	NA	NA	NA	--	--	2.18	--
**Leu718**	NA	NA	NA	NA	--	--	--	1.92
**Met793**	NA	NA	NA	NA	1.81	--	--	1.75
**Phe795**	NA	NA	NA	NA	--	2.09	2.00	--
**Asp800**	NA	NA	NA	NA	--	3.57	--	1.92
**Glu804**	NA	NA	NA	NA	--	2.05	1.87	--
**Arg841**	NA	NA	NA	NA	--	--	--	2.08

Supplementary Figure Legend

Supplementary Fig. S1a. ^{1}H NMR spectrum of a representative hybrid from the imatinib-Pt series (**1a**). The nitrogen labeled with an asterisk on the pyrimidine ring of imatinib binds with Pt to form the hybrid compound.

Supplementary Fig. S1b. 2D ^{1}H-^{13}C HSQC spectrum of **1a**.

Supplementary Fig. S1c. Delayed H-H COSY (coupling experiment) of **1a**.

Supplementary Fig. S1d. ^{14}N NMR spectrum of **1a** (top) and imatinib (bottom). For imatinib, the relatively broad peak at 286 ppm ($W_{1/2}$ = 48 Hz) in the pyridine nitogen region was assigned to the quinazoline nitrogens of the molecule. Downfield shift of the pyridine nitrogen (N_{py}) peak was observed after coupling to platinum. The sharper peak ($W_{1/2}$= 12-15 Hz) can be explained by the highly symmetric environment of the nitrogen after coupling to platinum.   In the inset, the spectrum of Pt-Imatinib hybrid was enlarged and two peaks (Pt-Npyridine and N-pyridine not coupled to Pt) were observed, indicating the Pt-imatinib hybrids were formed through a Pt-N bond.

Supplementary Fig. S1e. Mass spectrum of **1a**. The characteristic isotopic pattern of Pt is observed.

Supplementary Fig. S1f. ^{1}H NMR spectrum of a representative hybrid from the erlotinib-Pt series (2**b**).

Supplementary Fig. S1g. 2D ^{1}H-^{13}C HSQC spectrum of **2b**.

Supplementary Fig. S1h. NOE coupling experiment comparing erlotinib and **2b**.

Supplementary Fig. S1i. 1H NMR spectrum and 2D ^{1}H-^{13}C HSQC of erlotinib (starting material for **2b**) for comparison.

Supplementary Fig. S1j. Mass spectrum of **2b**. The characteristic isotopic pattern of Pt is observed.

Supplementary Fig. S1k. 1H NMR spectrum and 2D ^{1}H-^{13}C HSQC of **3a**.

Supplementary Fig. S2. HPLC chromatograms showing the gradual release of erlotinib from the hybrid **2c** upon incubation in intracellular compartment mimicking culture medium

supplemented with 5 mM GSH.

Supplementary Fig. S3. TREE*spot* compound profile data (DiscoveRx) summarizing the kinase inhibition of 132 oncogenic kinases by (S3a) erlotinib and **2a** (cisplatin-erlotinib), and (S3b) vandetanib and **3b** (oxaliplatin-vandetanib). Kinase inhibition profiling was performed by the KINOME*scan* service (DiscoveRx). The inhibition of different kinases is expressed as percentage of control and it is labeled in circle. The bigger the circle the greater is the inhibition effect.

Supplementary Fig. S4. *In vitro* nephrotoxicity of clinically used Pt anticancer drugs and the new Pt-TKI hybrids in the normal porcine kidney epithelial cell line LLC-PK1. Toxicity was evaluated by the release of lactate dehydrogenase (LDH) from drug-treated cells. The no treatment sample represents the spontaneous LDH release from cell culture. All Pt-TKI hybrids were tested at 100 μM. No Tx = no treatment; Oxa = oxaliplatin; Carbo = carboplatin.

Supplementary Fig. S5. Binding conformation of a representative Pt-erlotinib hybrid (**2a**) with EGFR bearing the secondary EGFR mutation. While steric hindrance from the T790M gatekeeper mutation is expected to affect the binding of erlotinib to EGFR, the binding of **2a** to EGFR T790M is likely less affected. **2a** was also found to form additional H-bond to D800 of the kinase domain of EGFR.

Supplementary Fig. S6. Cell-free kinase inhibition assay. Recombinant EGFR protein (wild type, L858/T790M, L858R/T790M/D800L) prepared with a baculovirus expression system was used in the Kinase-Glo luminescent kinase assay (Promega) in the presence of erlotinib or **2a** (cisplatin-erlotinib).

Supplementary Fig. S7. Mass spectrometric analysis of Pt-5'GMP monoadduct formation from **2a**.
(a) Mass spectrum of **2a** reacting with 5'GMP. Only monoadduct was detected. The peak at m/z = 985 corresponds to the monoadduct with +1 charge whereas the one at m/z = 493 corresponds to the monoadduct with +2 charges. The m/z region corresponding to the possible di-adduct (m/z = 955) is enlarged and shown in the small figure on the right. No Pt-5'GMP diadduct was detected for **2a**.
(b) Mass spectrum of cisplatin reacting with 5'GMP. Both Pt-G monoadduct (left) and Pt-GG diadduct (right) were formed.

1H NMR of **1a**

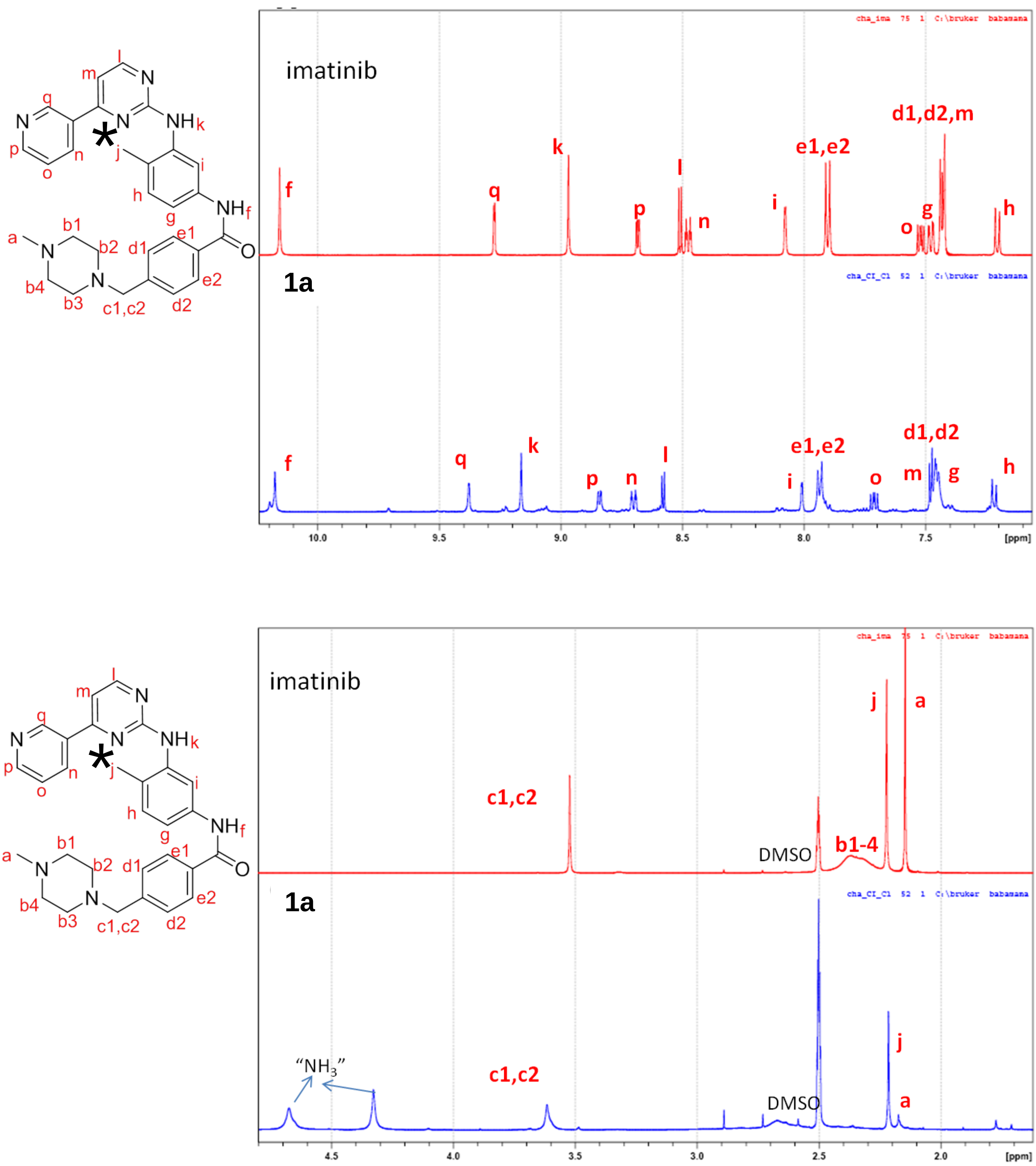

Supplementary Fig. S1a

2D 1H-13C HSQC of **1a**

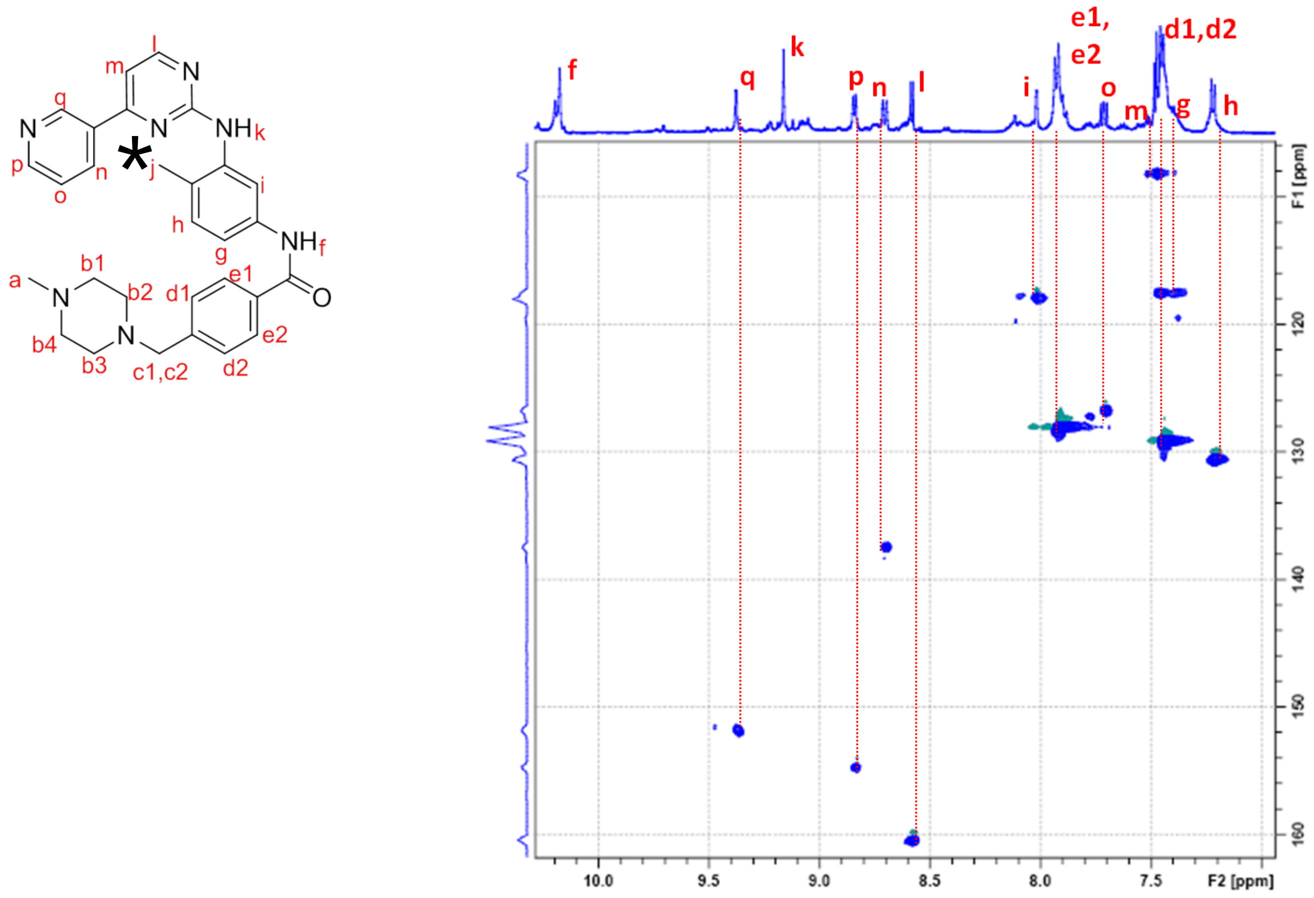

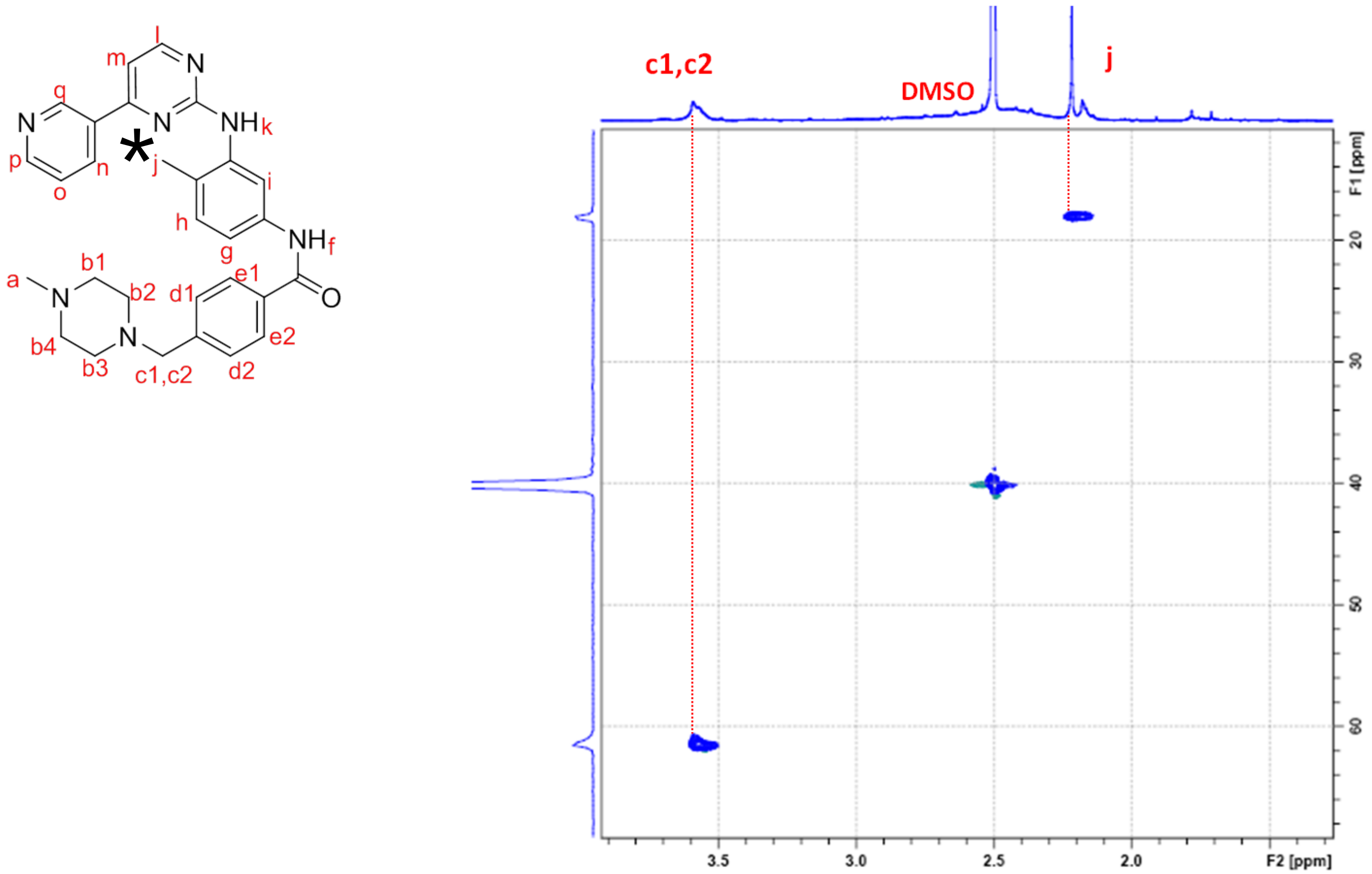

Supplementary Fig. S1b

Delayed H-H COSY of **1a**

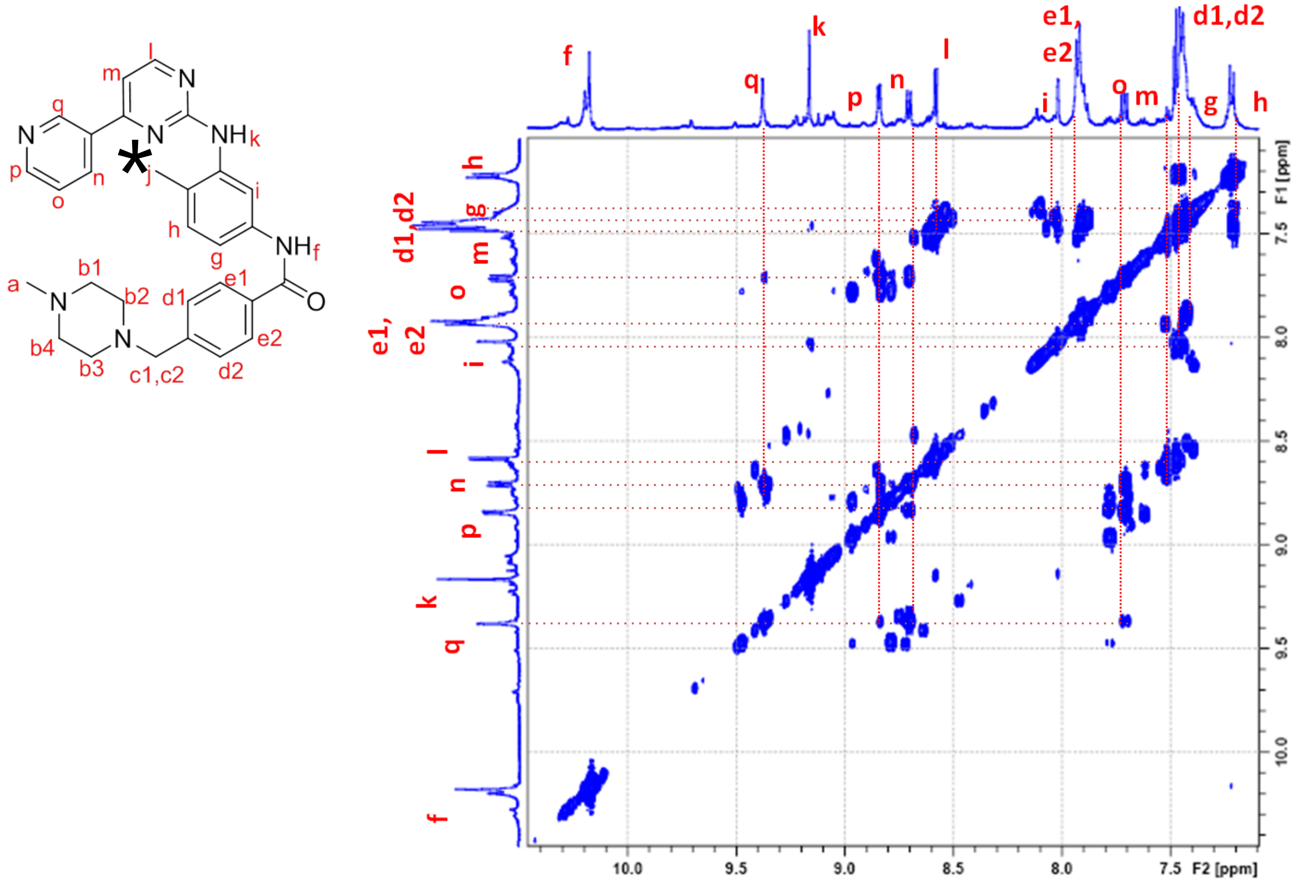

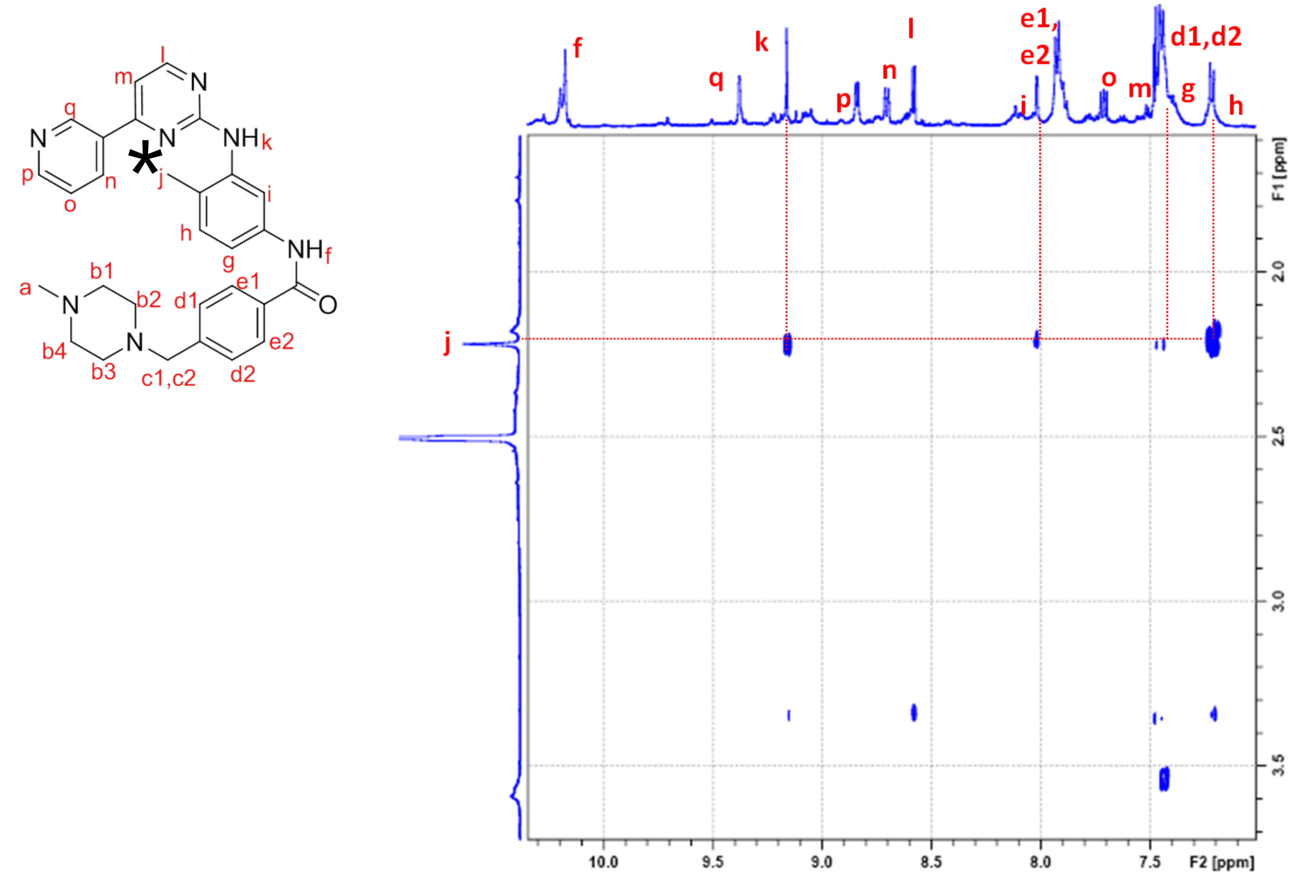

Supplementary Fig. S1c

14N NMR of **1a**

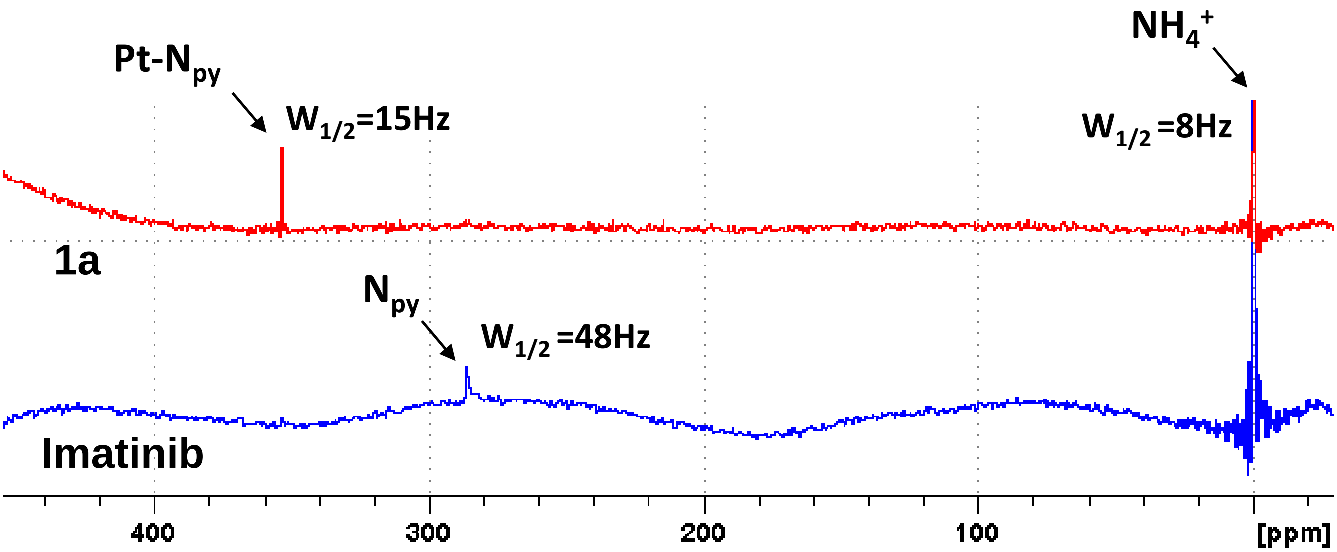

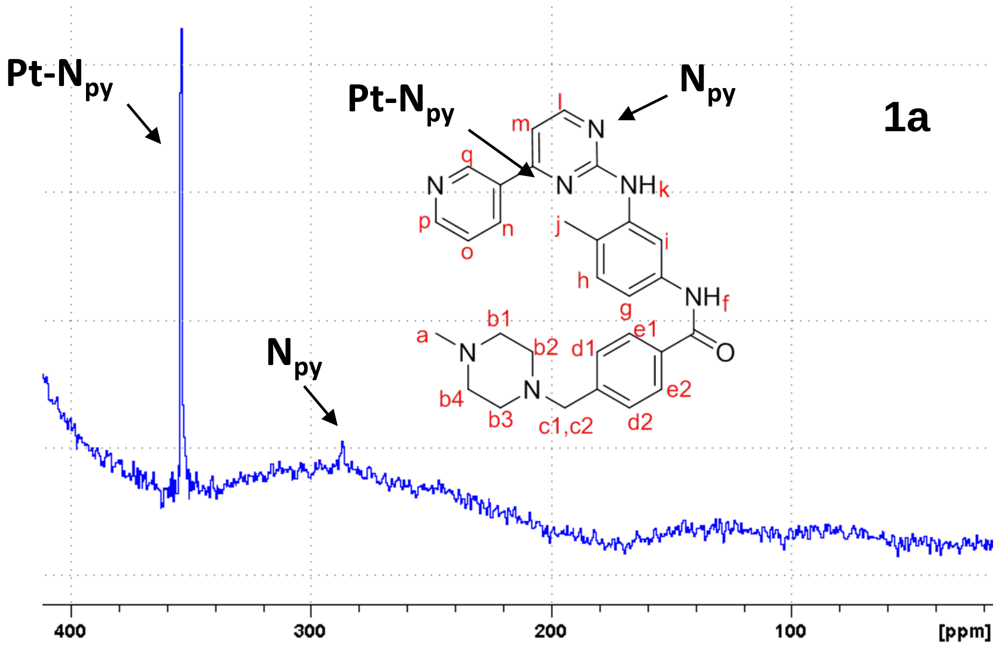

Supplementary Fig. S1d

Mass spectrum of **1a**

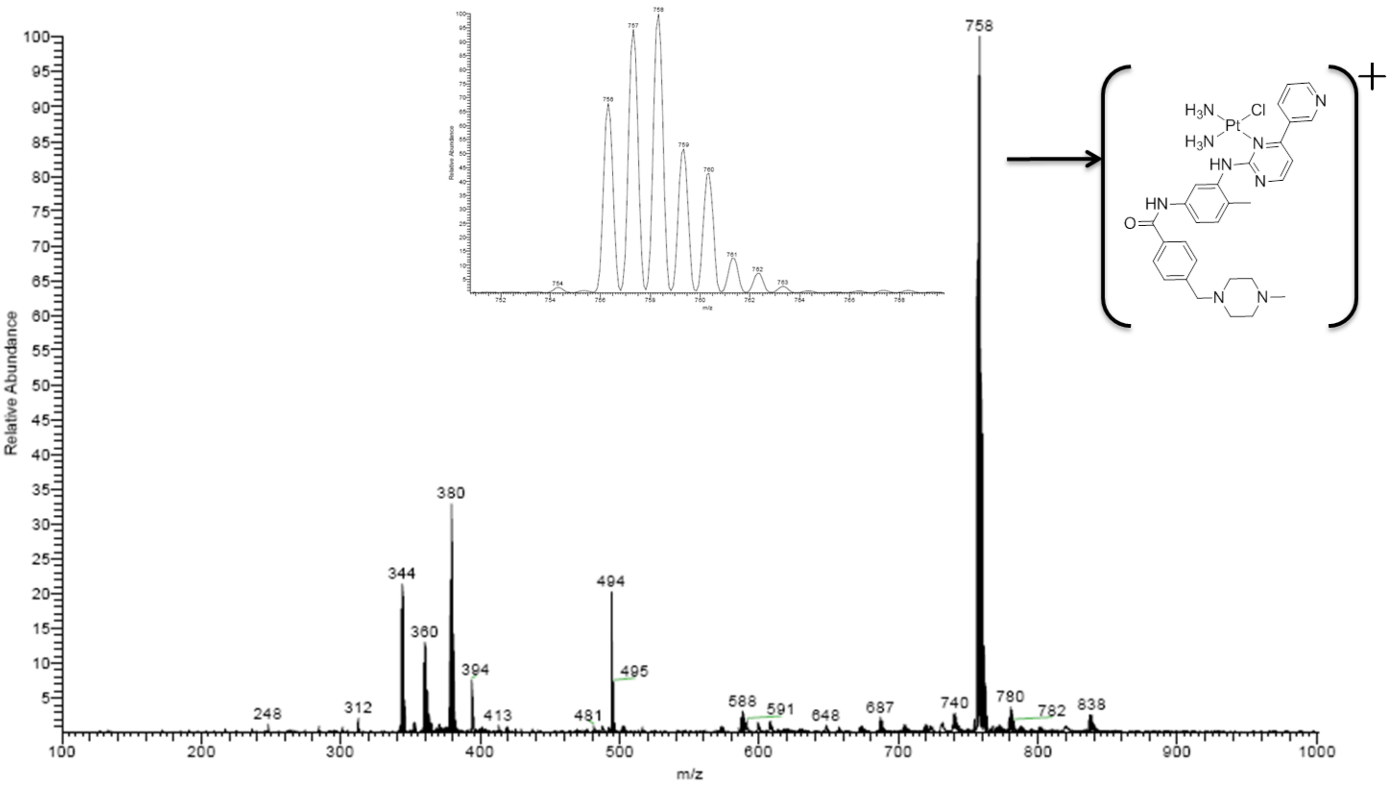

Supplementary Fig. S1e

1H NMR of **2b**

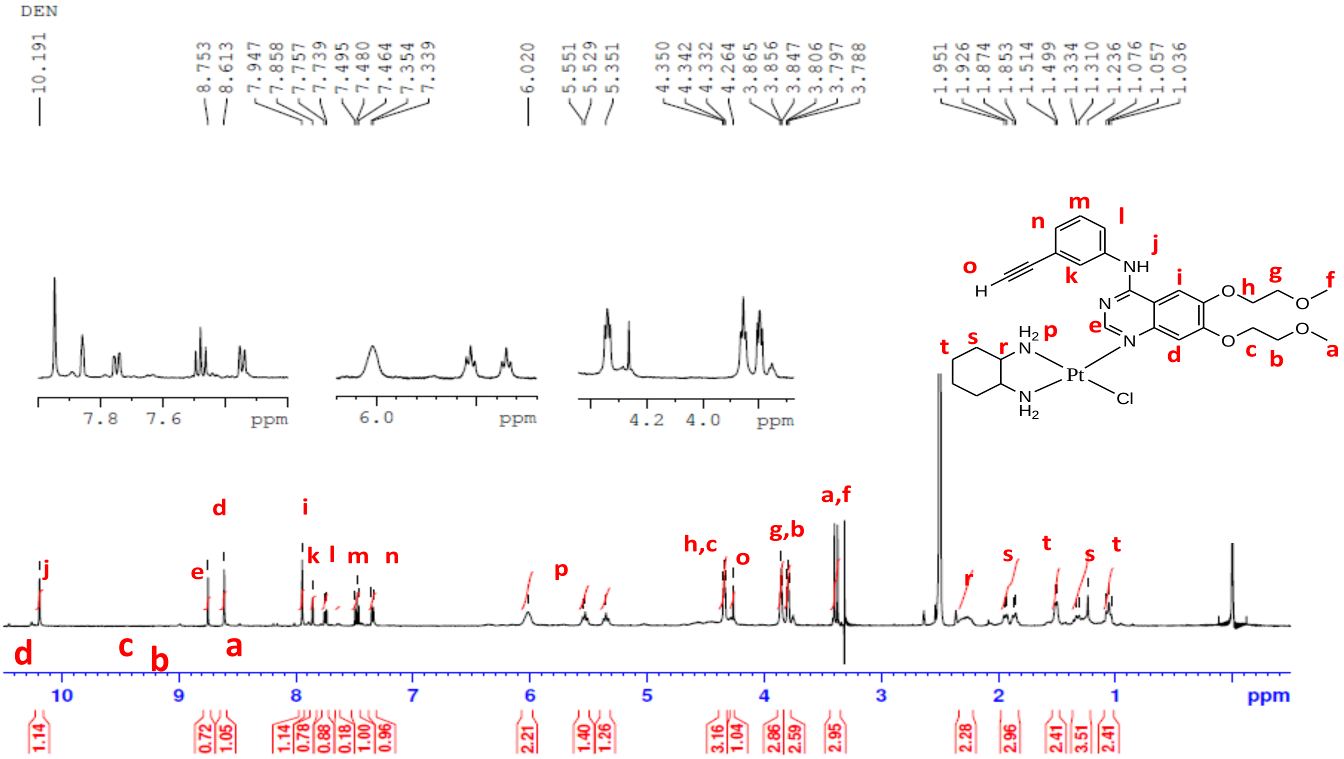

Supplementary Fig. S1f

2D 1H-13C HSQC of **2b**

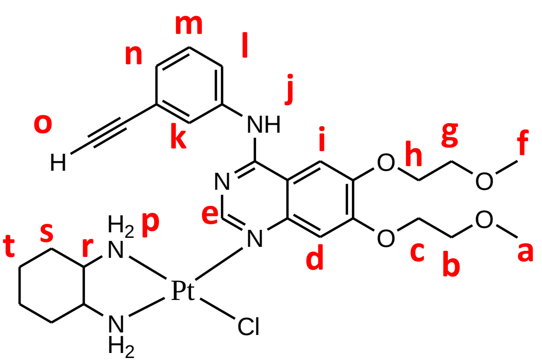

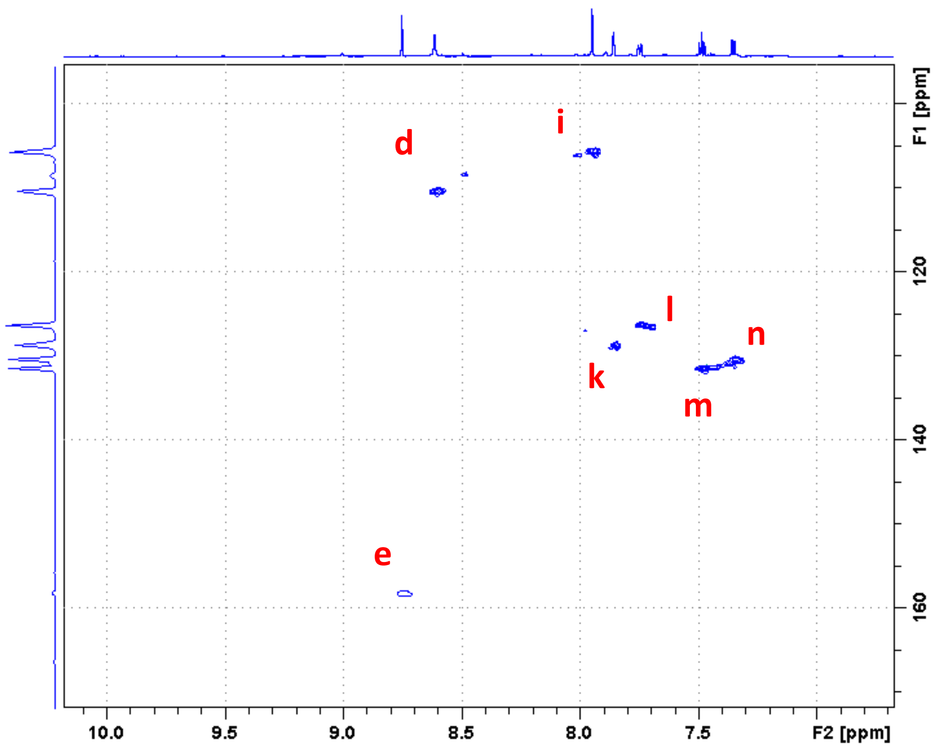

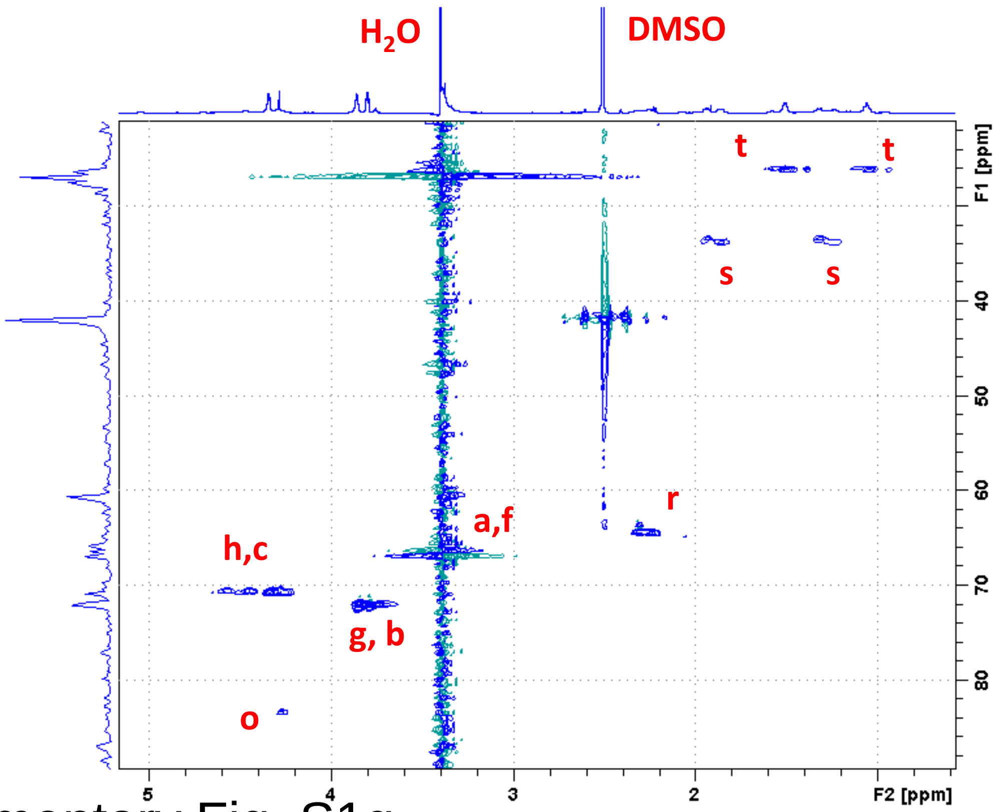

Supplementary Fig. S1g

NOE coupling between Hj and Hi peaks of erlotinib

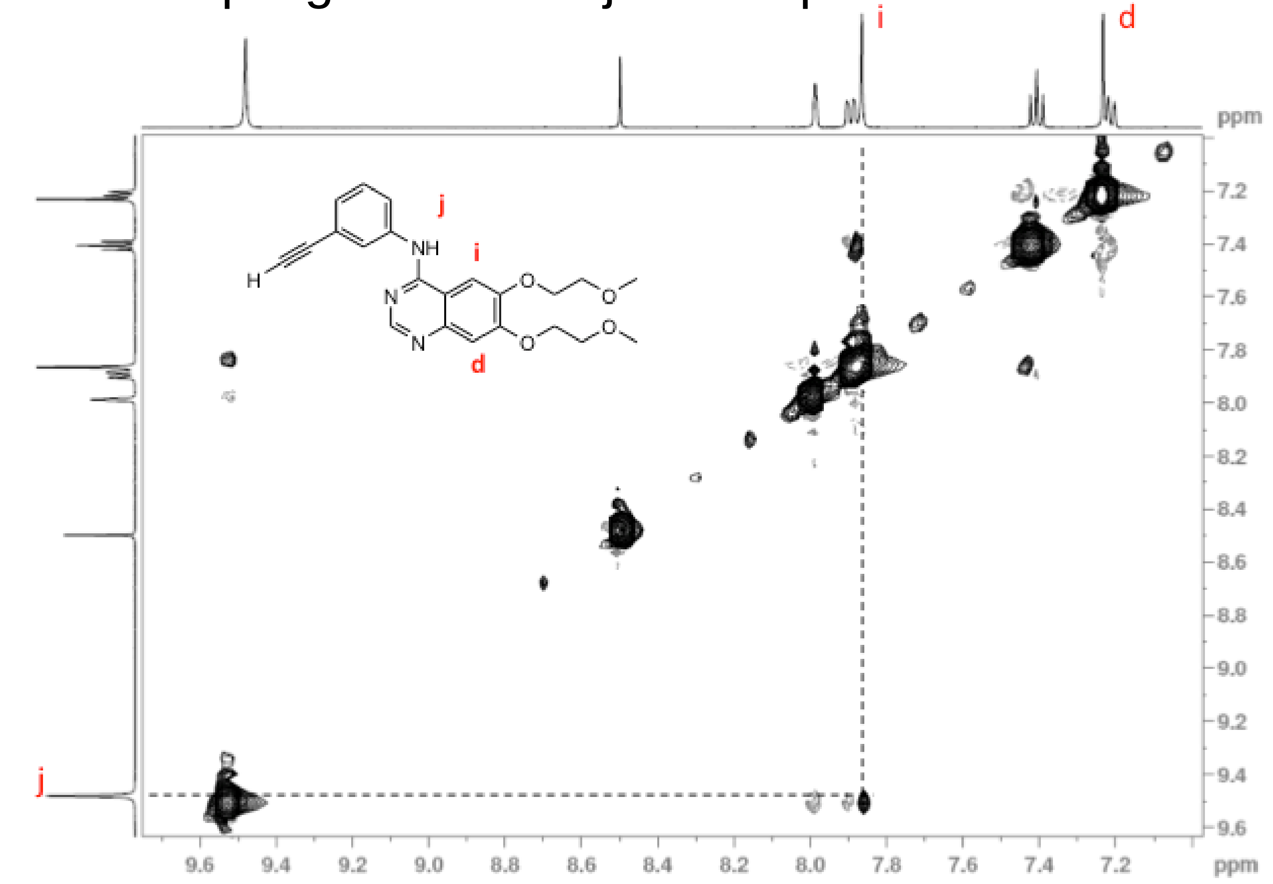

NOE coupling between Hj and Hi peaks of **2b**

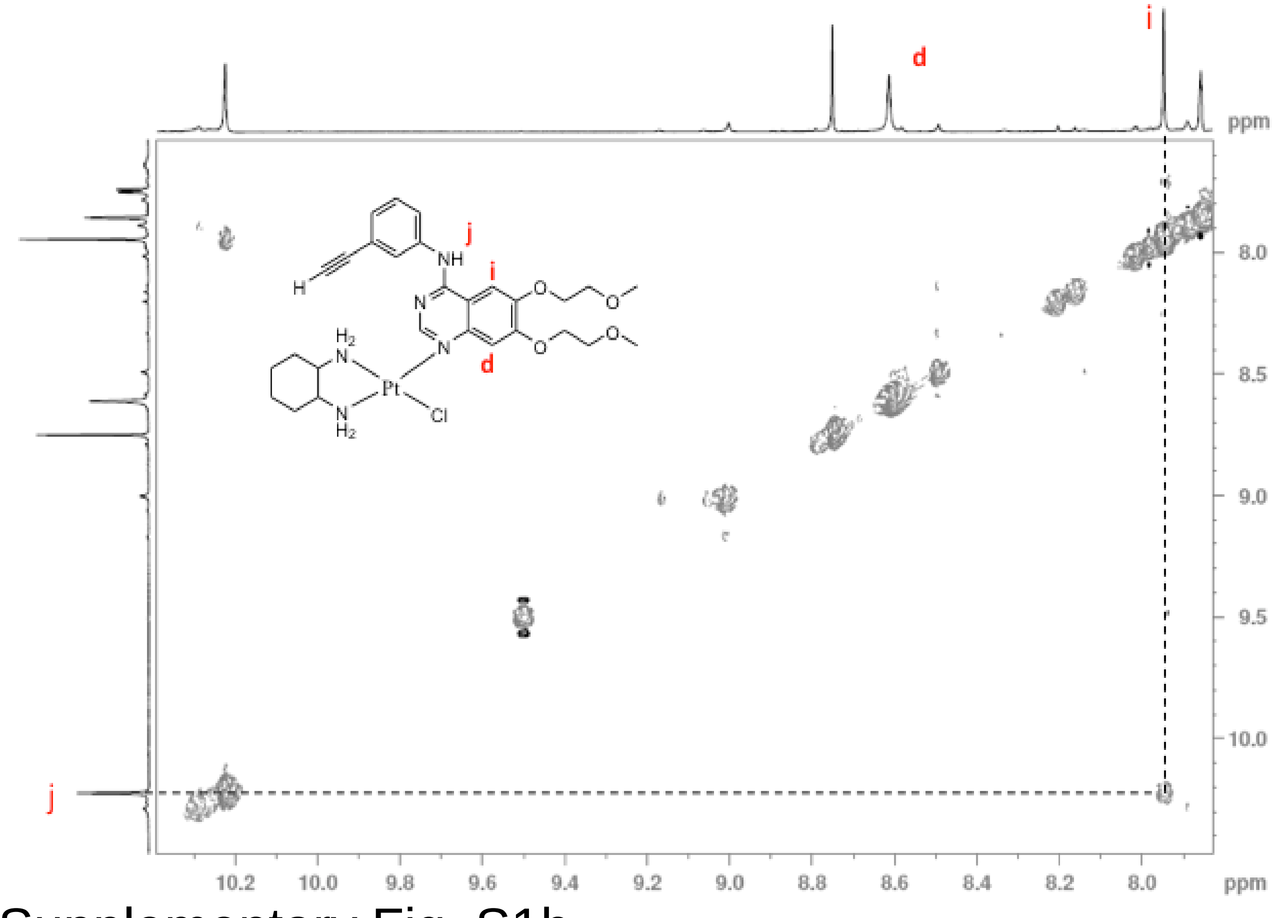

Supplementary Fig. S1h

1H NMR of **erlotinib** (for comparison)

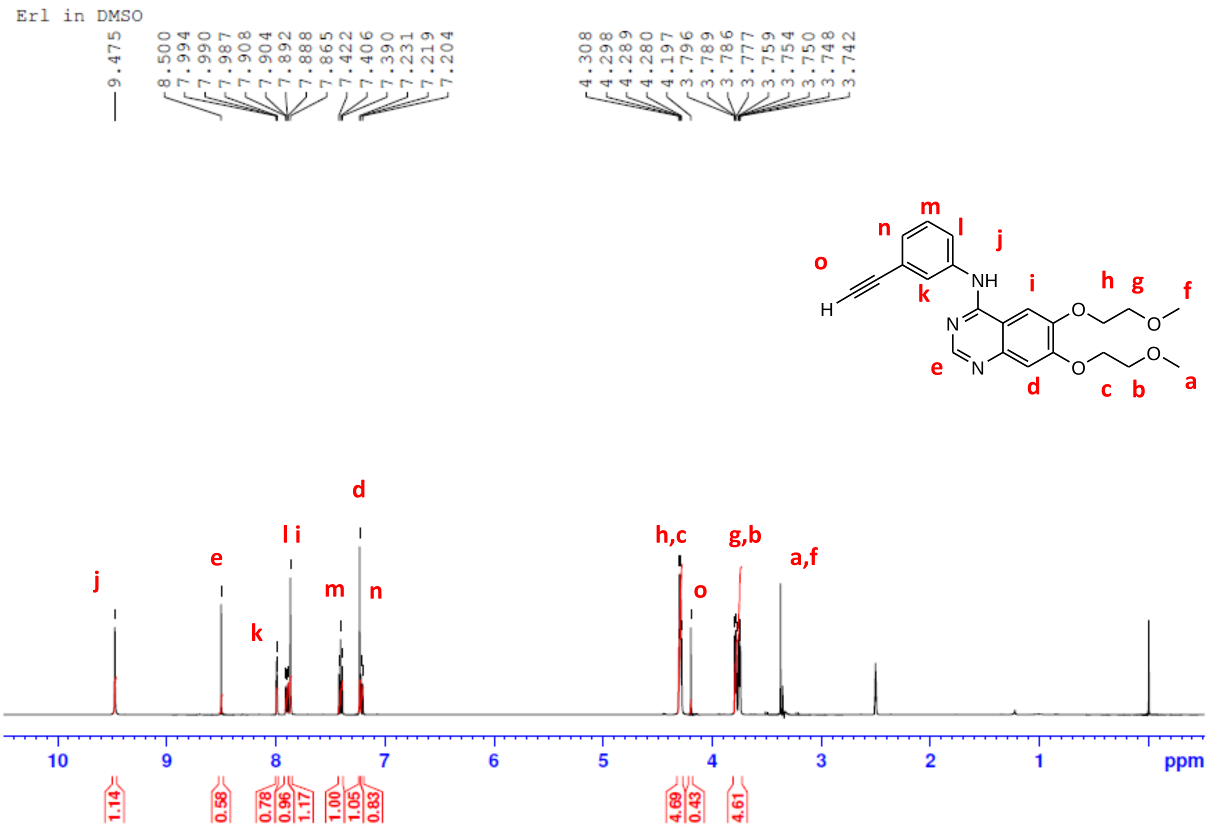

2D 1H-13C HSQC of **erlotinib**

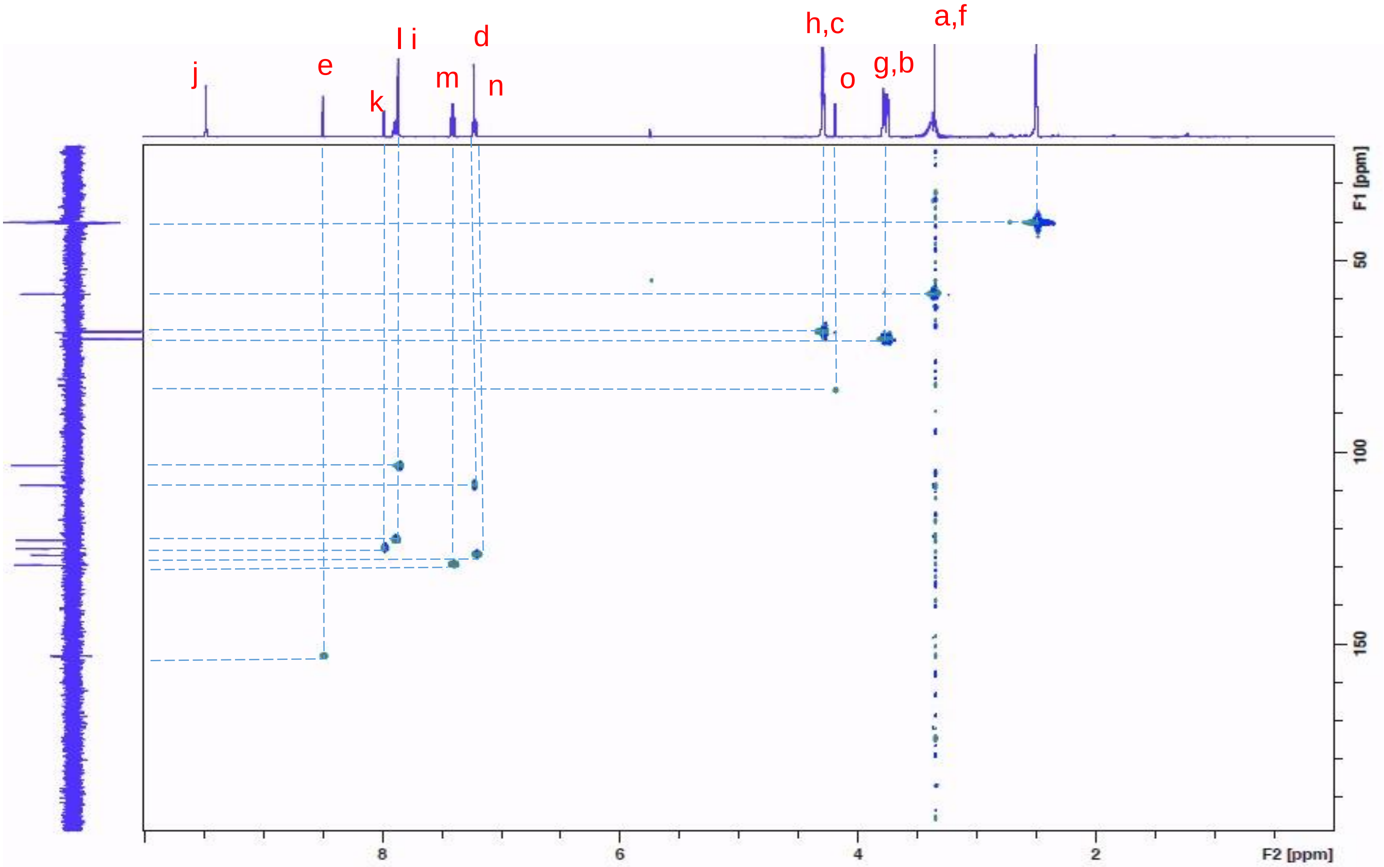

Supplementary Fig. S1i

Mass spectrum of **2b**

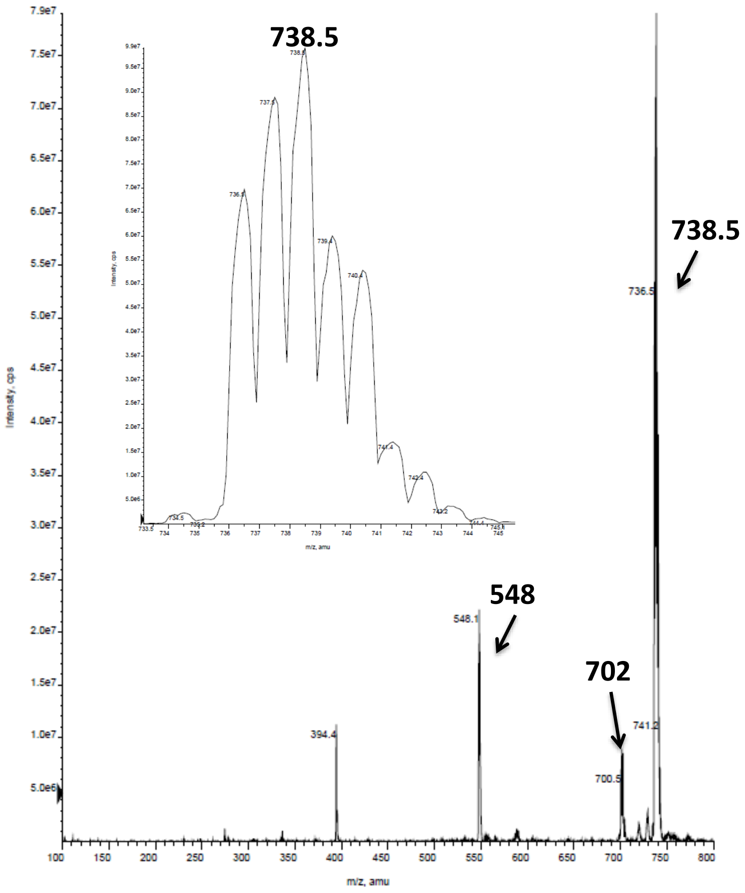

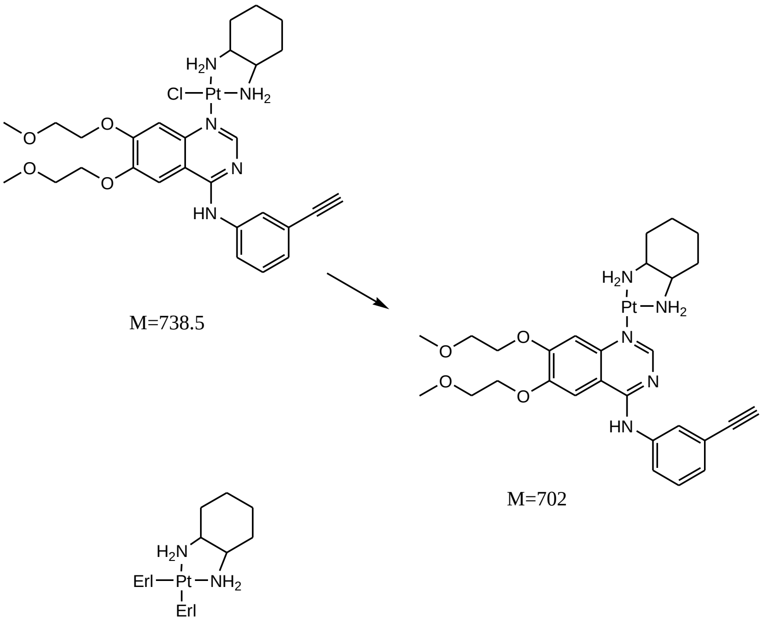

M=738.5

M=702

M=1095

Supplementary Fig. S1j

1H NMR of **3a**

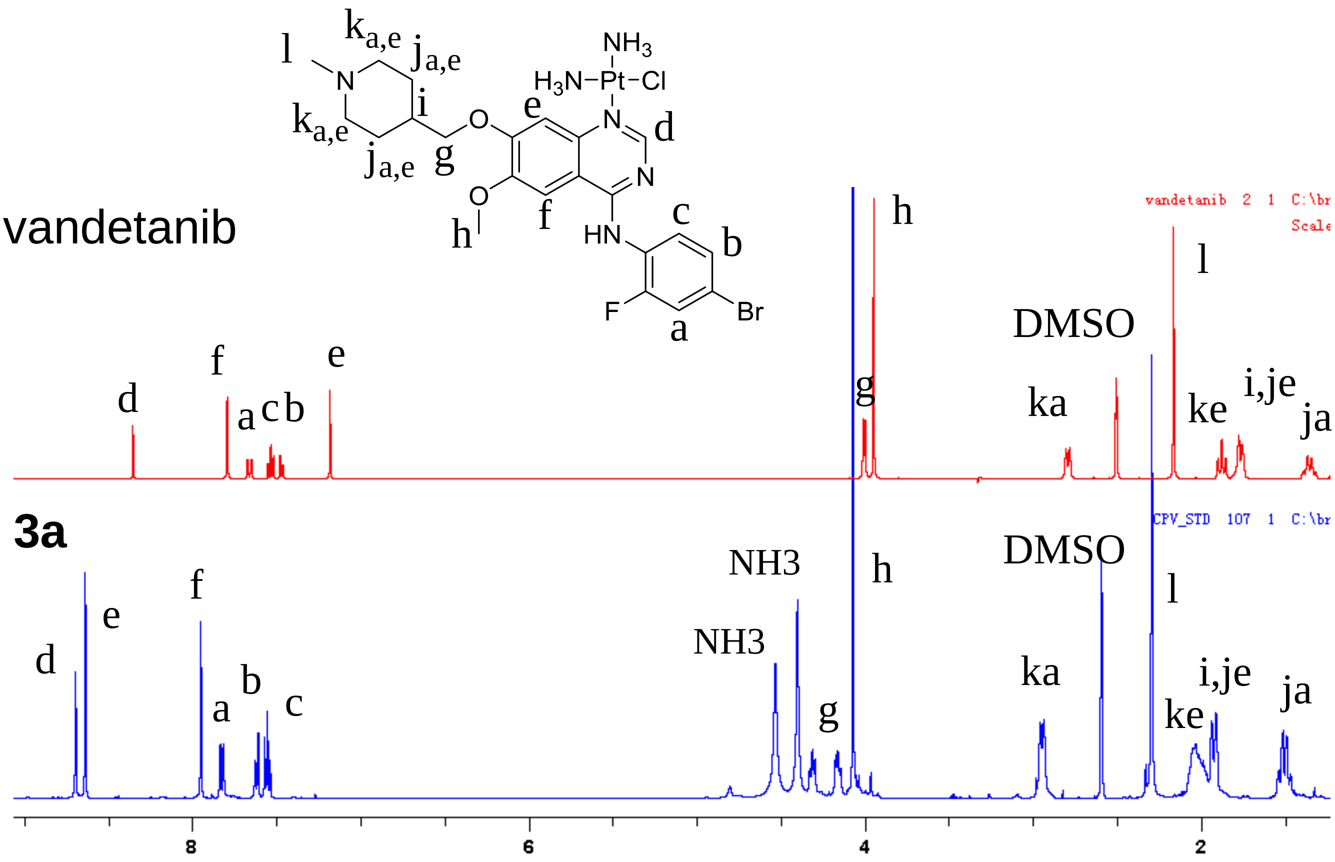

2D 1H-13C HSQC of **3a**

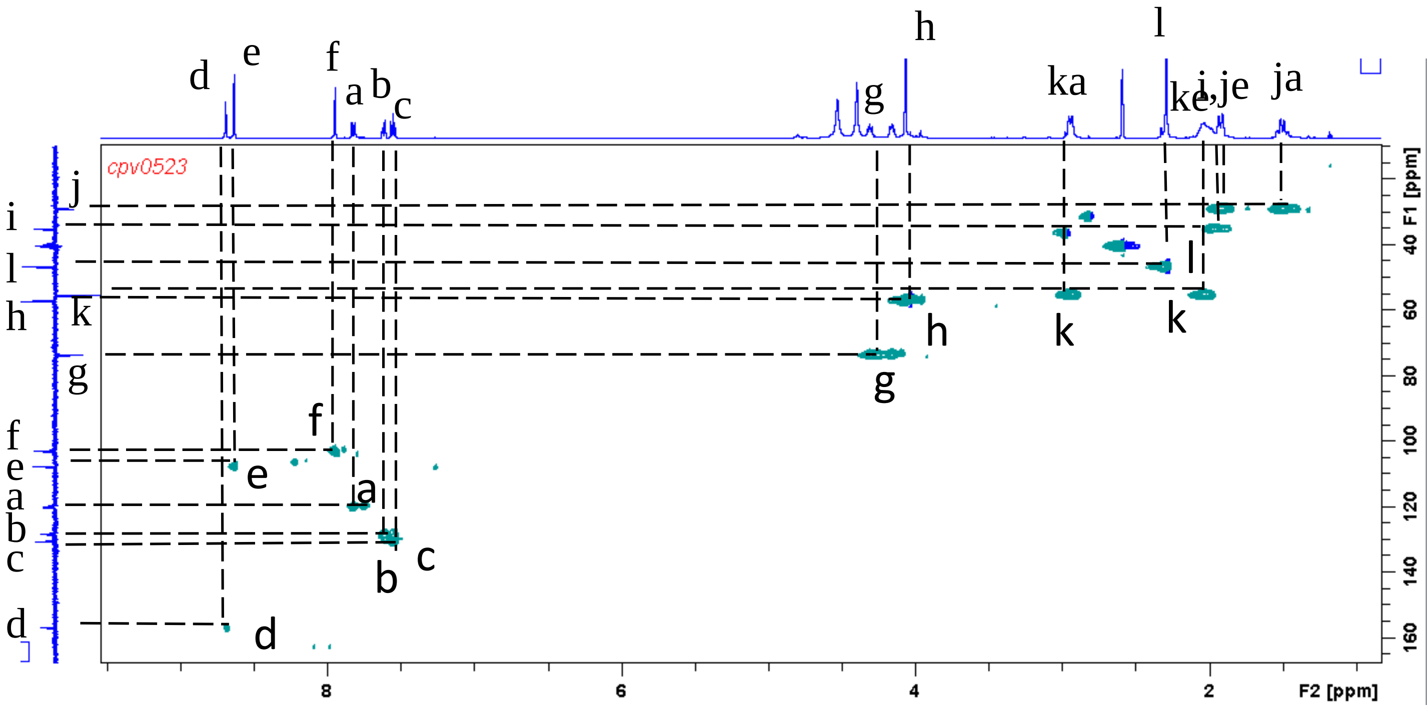

Supplementary Fig. S1k

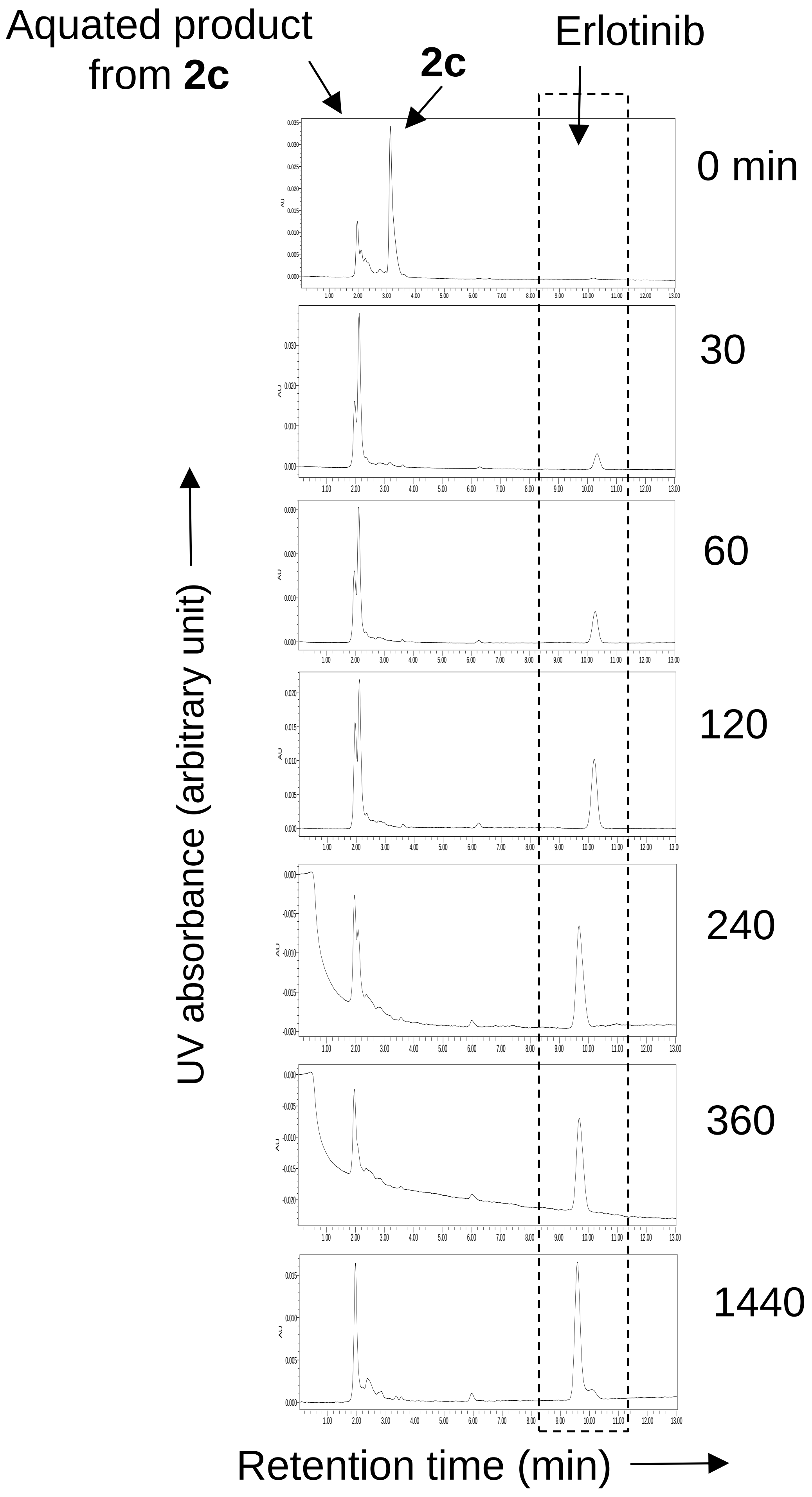

Supplementary Figure S2

Erlotinib

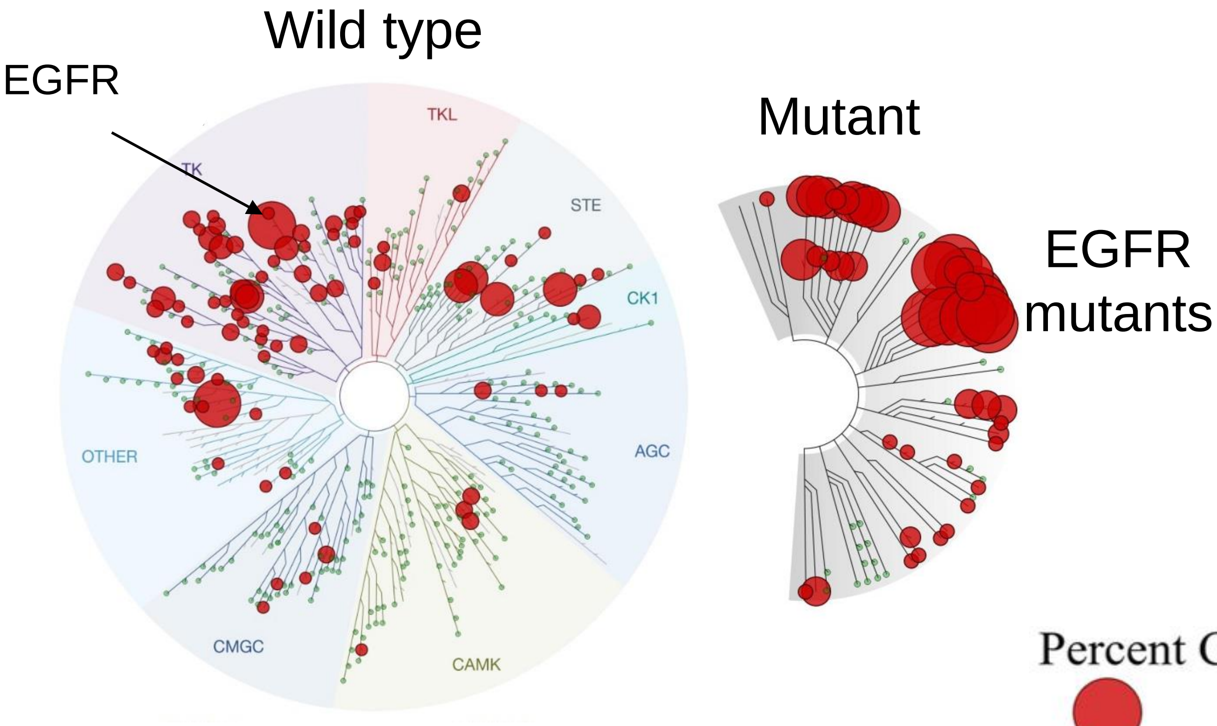

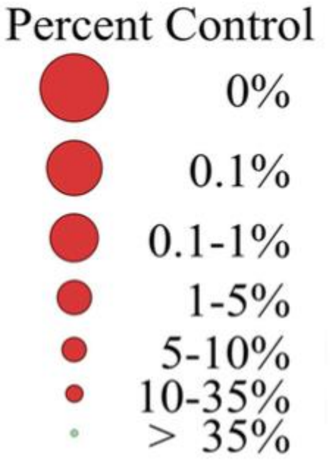

Cisplatin-erlotinib (**2a**)

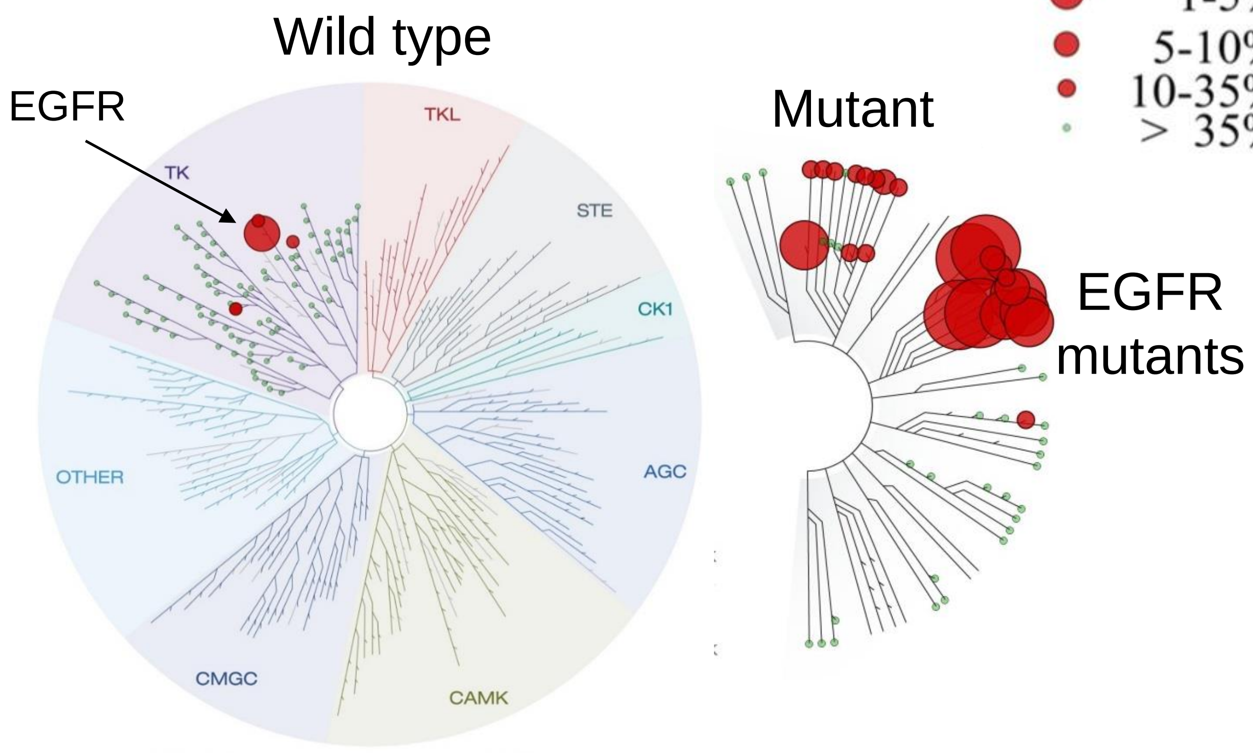

Supplementary Figure S3a

Vandetanib

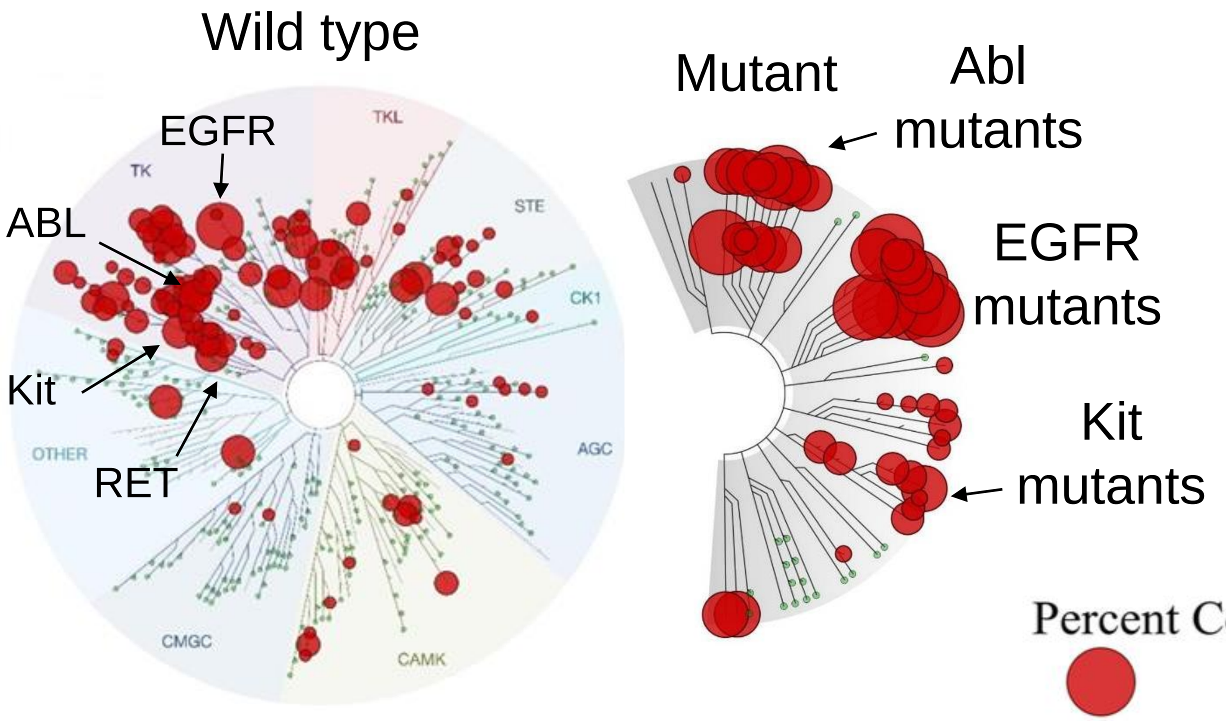

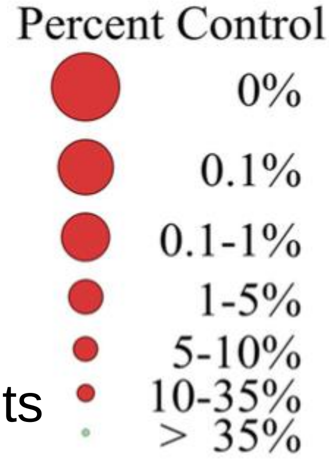

Oxaliplatin-vandetanib (**3b**)

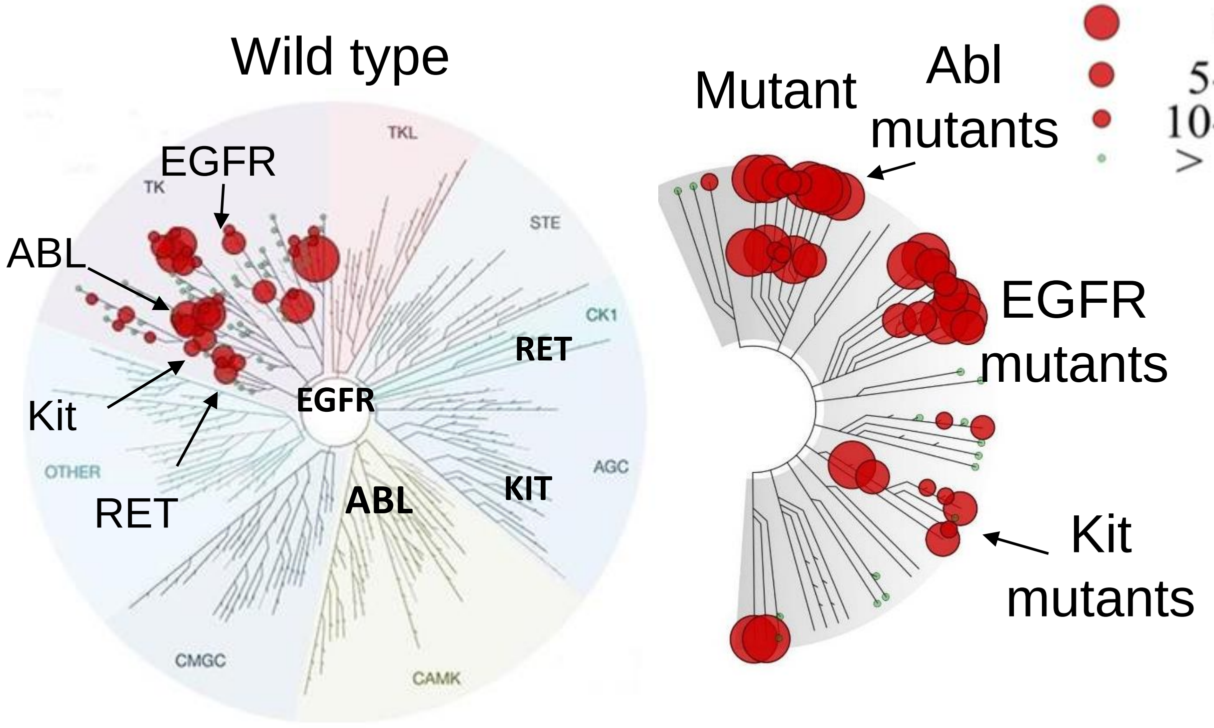

Supplementary Figure S3b

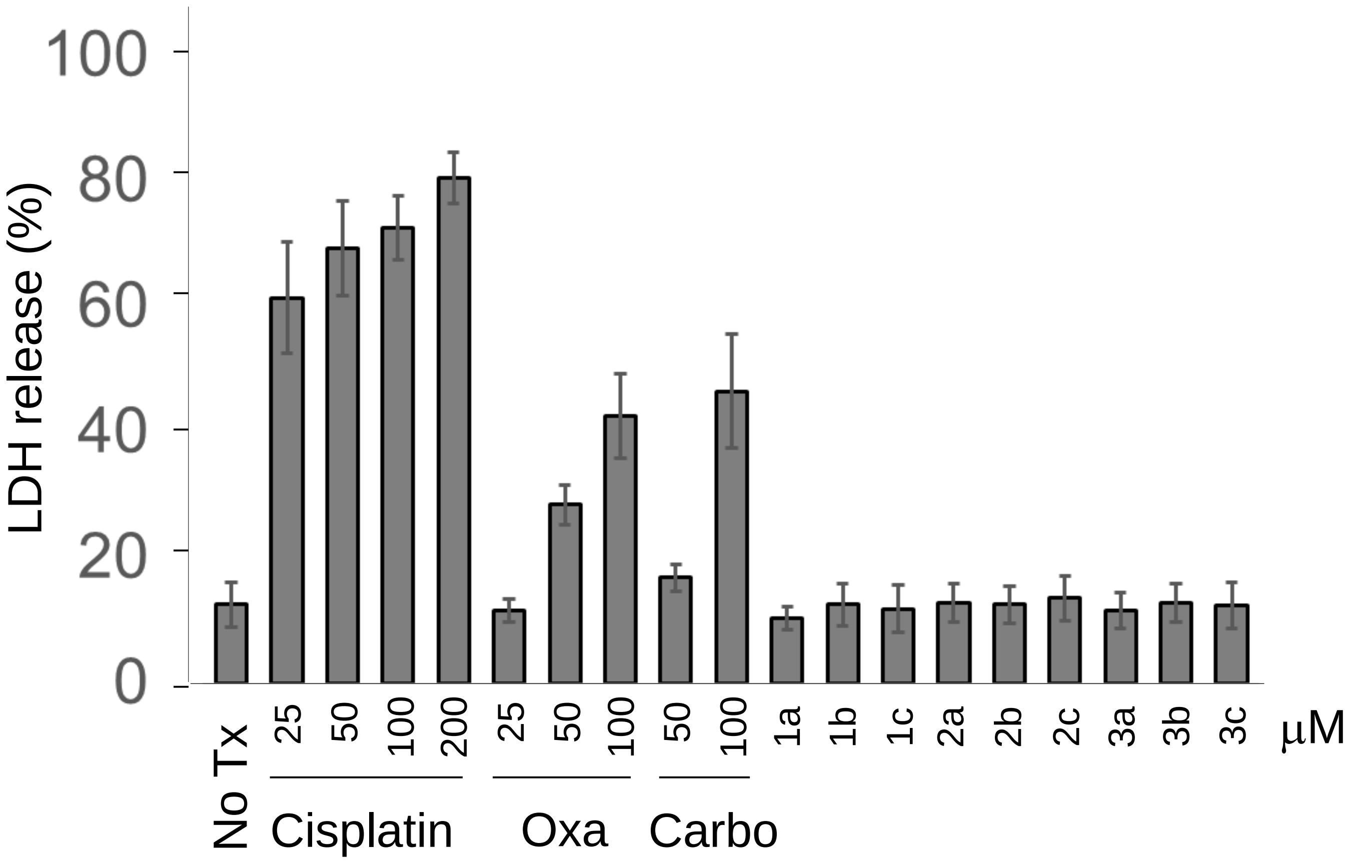

Supplementary Figure S4

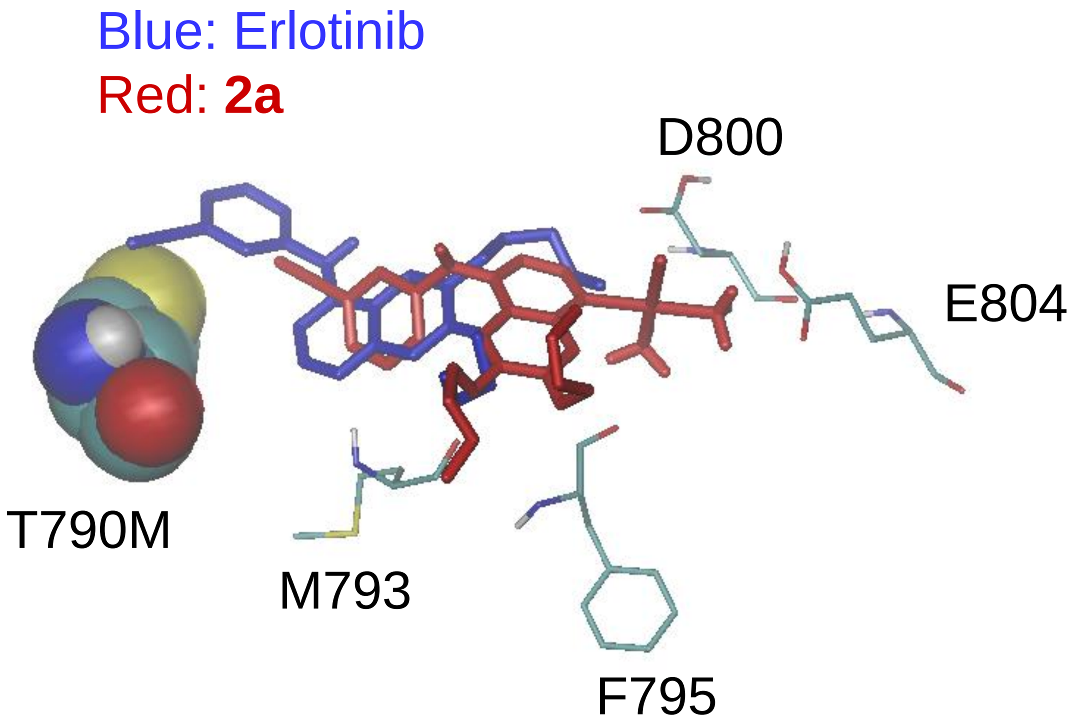

Supplementary Figure S5

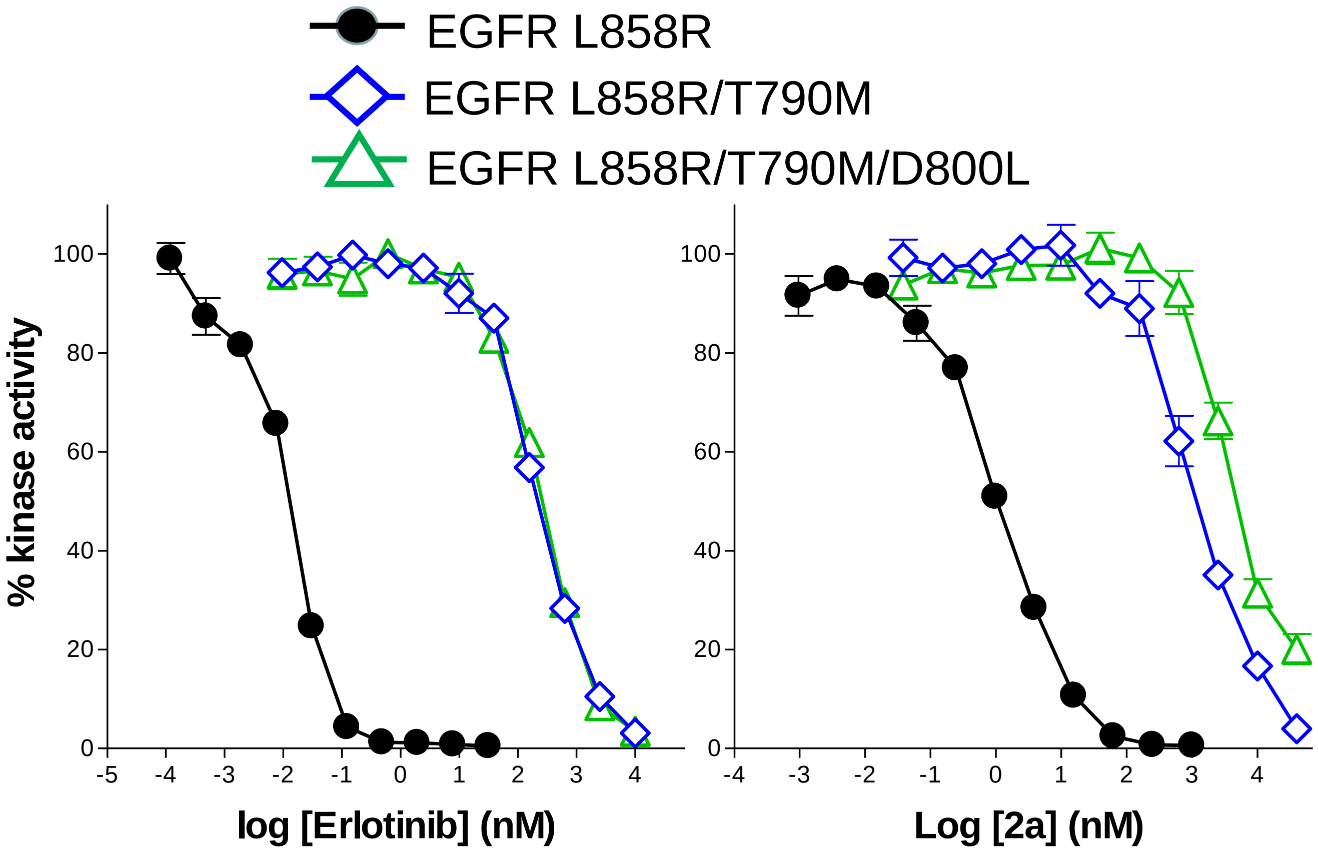

Supplementary Figure S6

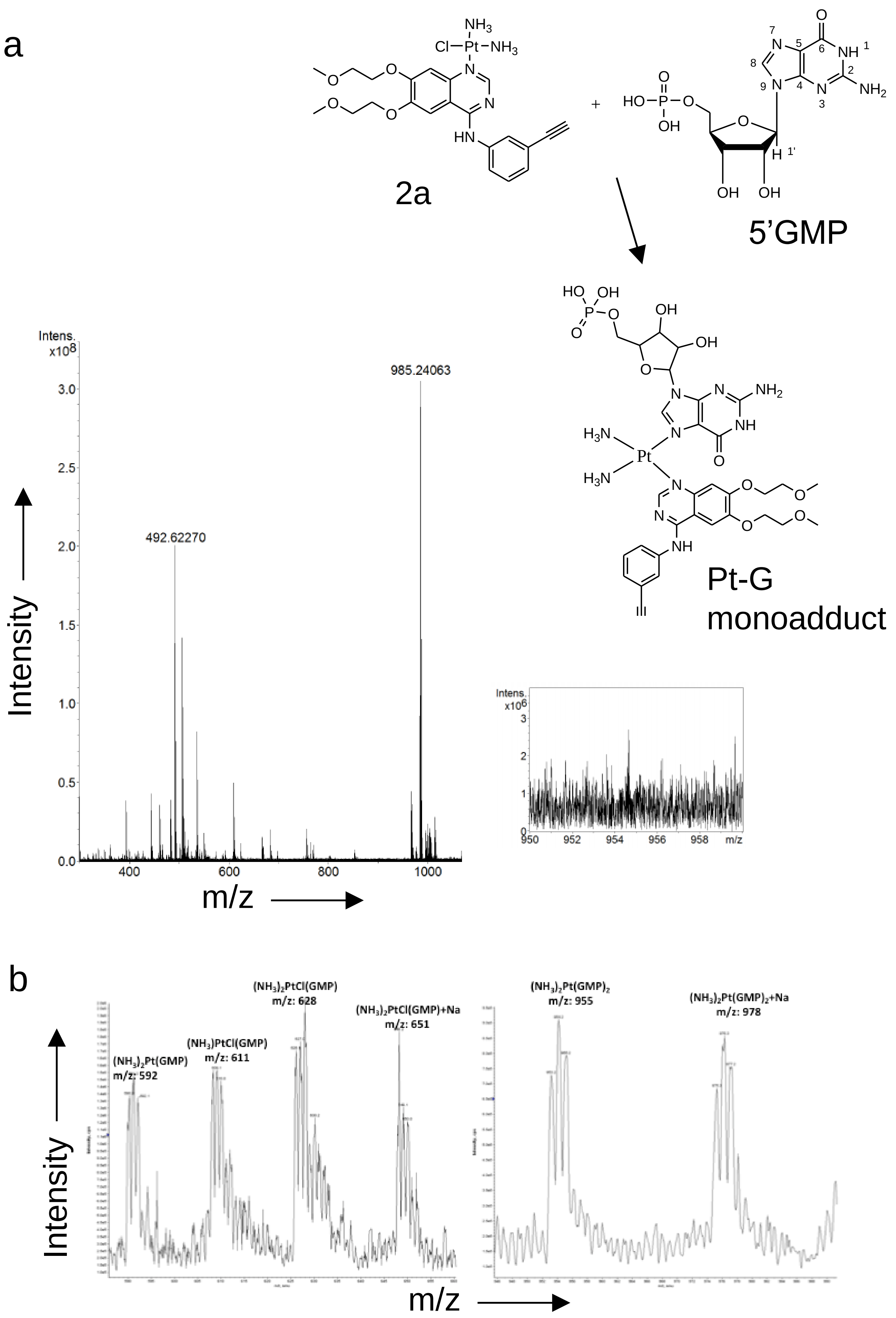

Supplementary Figure S7
